# Investigating the Components of Body Image Disturbance Within Eating Disorders

**DOI:** 10.3389/fpsyt.2019.00635

**Published:** 2019-09-18

**Authors:** Mark Carey, Catherine Preston

**Affiliations:** Department of Psychology, University of York, York, United Kingdom

**Keywords:** eating disorders, body image, multisensory integration, moving rubber hand illusion, implicit body satisfaction

## Abstract

Body image disturbance has been highlighted as a common characteristic within the development and maintenance of clinical eating disorders (EDs), represented by alterations in an individual’s bodily experience. However, whilst the perceptual stability of the sense of body ownership has been investigated in ED patients, the stability of the sense of body agency in those with ED is yet to be examined. Therefore, body ownership and body agency were investigated using the moving rubber hand illusion, alongside measures of explicit and implicit body satisfaction. Furthermore, with evidence demonstrating a direct link between perceptual and cognitive-affective components of body image in the healthy population, the relationship between measures of body perception and body satisfaction was investigated. Results showed that both ED and healthy individuals displayed a similar subjective experience of illusory ownership and agency towards the fake hand, following voluntary movement. However, whilst both groups initially overestimated their own hand width prior to the illusion, the ED group displayed a significant reduction in hand size estimation following the illusion, which was not matched to the same degree in healthy individuals. In addition, ED individuals displayed a significantly lower body satisfaction compared with healthy females, on both an explicit and implicit level. Such implicit outcomes were shown to be driven specifically by a weaker association between the self and attractiveness. Finally, a significant relationship was observed between specific perceptual measures and implicit body satisfaction, which highlights the important link between perceptual and cognitive-affective components of one’s body image. Together, such findings provide a useful foundation for further research to study the conditions in which these two components relate with regard to body image and its disturbance, particularly in relation to the prognosis and treatment of EDs.

## Introduction

A common hallmark in the development and maintenance of clinical eating disorders (EDs) is a disturbance in body image (1), which refers to distortions or alterations in the way in which an individual experiences his/her body shape or weight (2). Body image disturbance is argued to be a multidimensional construct, which is commonly divided into two key components (3). The perceptual component denotes issues in estimating one’s own body size and dimensions, with evidence that, at a group level, ED individuals typically overestimate the size of their own body significantly more than healthy individuals (4, 5). Additionally, the cognitive-affective component is associated with negative attitudes and emotions towards one’s own body, commonly displayed by extreme feelings of body dissatisfaction amongst ED patients (3, 6). Indeed, research has suggested that ED individuals lack a self-serving body image bias that is typically observed in the healthy population, which reflects a highly biased positive perception to one’s own attractiveness relative to the perception from others (7). Importantly, such a self-serving bias in healthy individuals acts as a protective factor against poor mental health (8); therefore, the lack of such a bias is likely to have a negative effect towards one’s body satisfaction amongst EDs.

Historically, research has predominantly focused on the cognitive-affective component of body image disturbances within EDs (3, 9), with treatment programmes commonly targeting dysfunctional cognitions and emotions relating to the body (8, 10, 11). However, more recent research suggests that such distorted cognitions may be influenced by an inaccurate perceptual experience of the body (12, 13), which remains comparably less understood amongst EDs (14). Indeed, evidence has shown that clinical outcomes are poorer amongst those who report greater misperception of their body (14–16). Moreover, the perceptual component of body image disturbances in EDs has primarily been investigated using visual size estimation tasks (3, 4, 17). However, recent neuroscientific research has revealed higher-order perceptual disturbances amongst EDs within multiple sensory domains, including tactile perception (18, 19), proprioception (20,), interoception (22, 23), and the integration of multiple sensory signals (13, 24). Therefore, it is important that research investigates how ED individuals process multisensory body information and the role this might play within the perceptual component of body image disturbances.

Disturbances in the integration of sensory information have been observed amongst ED patients using multisensory body illusions (24). The most studied of these paradigms is the rubber hand illusion (RHI), in which individuals typically experience ownership over a fake rubber hand when it is stroked synchronously with their own hand, which is hidden out of view (25). Crucially, ED patients have been shown to display a greater sense of ownership towards the fake hand compared with healthy controls (HCs) during the RHI, following both synchronous (illusion) and asynchronous (control) conditions, with susceptibility to the illusion positively associated with ED psychopathology (24). Such findings suggest that ED individuals display a greater reliance towards visual body information, which dominates proprioceptive sensory input during body ownership. More recent work has provided corroborative evidence, with induction to the RHI also shown to improve initial overestimation of hand size amongst patients (13), which highlights that such malleability observed in patients’ body representation can be developed to a more accurate estimation of one’s own body size (13, 26). Taken together, the above evidence underlines the importance of researching perceptual disturbances of body image in EDs from a multisensory perspective (27), with improvements in the perceptual accuracy of one’s own body dimensions likely to act as a protective factor against relapse if coping strategies designed to address cognitive-affective components of body image were to break down (28).

A component that is intimately linked with body ownership in contributing towards one’s coherent body representation is the sense of agency, which refers to the experience of authorship over an active, volitional bodily movement (29–31). Such a sense of control over one’s motor actions is essential in contributing towards one’s bodily experience and interaction with the external environment (29). Indeed, disturbances in the sense of agency have been implicated as an important feature within numerous psychiatric disorders (32, 33). Importantly, whilst research has shown that ED patients display alterations in the execution of body-scaled action with regard to unconscious sensorimotor aspects of body representation (34–36), the conscious sense of agency has not been directly investigated within EDs, particularly how alterations in this component may play a role within body image disturbances. An existing experimental paradigm that measures the sense of body ownership and agency is the moving rubber hand illusion (mRHI) (37, 38), which extends upon the RHI by introducing active, volitional movement to a fake model hand. In a similar manner to the classic RHI, synchronous movements typically elicit a strong sense of ownership towards the fake hand, but also a sense of agency, i.e. feeling of controlling the movement of the fake hand. Such feelings of agency are absent when voluntary movements are asynchronous with the movements of the fake hand. Therefore, the mRHI provides the opportunity to experimentally investigate the sense of body ownership and body agency and their relationship in contributing towards a coherent body representation.

With regard to the cognitive-affective component of body image disturbance, the most commonly used assessments of ED pathology in research and treatment include self-reports (e.g. clinical interviews, standardised questionnaires) that target explicit cognitions and behaviours (39). However, research has shown that such explicit measures alone may not accurately reflect an individual’s attitudes or behaviours towards certain concepts (40, 41), particularly amongst ED patients who can display denial towards the severity of their disorder (42). Therefore, it is clinically useful to supplement explicit body-related measures with implicit measures that are free from response bias. Implicit cognitive mechanisms are argued to play a key role in the pathology of EDs (44, 45) and could provide an insight into an ED individual’s disordered cognitions and behaviours that cannot be obtained from self-reports (43). A commonly used measure to assess implicit attitudes is the Implicit Association Test (IAT) (46), which is a computer-based reaction time task designed to measure the strength of automatic association between certain concepts (see *Methods* section for further details). Conceptually, it is argued that individuals typically pair target words more quickly with the category that is consistent with their own beliefs or cognitions (46). Therefore, the IAT provides the opportunity to tap into an individual’s implicit cognitions towards certain concepts, including the self.

Whilst many studies have used the IAT to measure implicit social attitudes (47), studies have also measured implicit attitudes and cognitions towards the self (48–51). Previous research has established a relationship between implicit body satisfaction with ED symptoms in healthy individuals (40, 52, 53). Moreover, previous studies have examined implicit attitudes towards body size (54–56) and food (57). However, to the authors’ knowledge, the present study is the first to investigate implicit body satisfaction using the IAT in an ED sample. Investigating implicit cognitions towards body satisfaction amongst ED individuals is important in understanding the multifaceted constructs that underlie body image disturbances (9), particularly how explicit and implicit cognitions relate to each other, which may have important implications for long-term recovery and relapse.

Taken together, the present study examines both perceptual and cognitive-affective components of body image in EDs, extending each with agency and implicit measures, respectively. First, given the intrinsic link between body ownership and agency towards a coherent body representation, it is hypothesised that the predicted instability in the sense of body ownership would also feed into instability towards the sense of body agency in ED individuals. Moreover, the effect of the illusion was investigated towards perceptual estimations of hand size. In line with previous research (13), it is predicted that ED individuals will show initial overestimation of their own hand size but improve their accuracy following the illusion, with HCs expected to display a stable estimation throughout. Second, it is predicted that lower explicit body satisfaction, which is expected to be displayed in ED individuals, would also extend to lower body satisfaction on an implicit level, compared with healthy females. Third, whilst it has been previously argued that perceptual and cognitive-affective alterations contribute independently towards body image disturbances (3), increasing research has highlighted a direct link between the body perception and the emotional body experience within healthy and clinical samples (53, 58–60). Therefore, the possible links between body perception and body satisfaction were investigated, in relation to the influence this may have in ED psychopathology. It is predicted that individuals with greater instability on perceptual multisensory illusion measures would display reduced scores on body satisfaction measures.

## Methods

### Participants

The present study received ethical approval from the NHS Health Research Authority (North East – York Research Ethics Committee; Project ID 199702); The Retreat Mental Health Care Centre, York (Research Governance Committee); *Beat* Eating Disorders Charity Research Ethics Committee; and the University of York Departmental Ethics Committee. The study was conducted in accordance with the Declaration of Helsinki, with all participants providing informed consent to take part.

Twenty-eight female participants with an ED diagnosis participated in the present study [mean age, 26.11 (SD, ± 11.69) years]. The ED group consisted of 19 individuals with a diagnosis of anorexia nervosa (AN), 2 with a diagnosis of bulimia nervosa (BN), 2 with a diagnosis of binge eating disorder (BED), and 5 with other specified feeding or eating disorder (OSFED). Of the above sample, 5 participants were recruited as inpatients *via* The Retreat, York (Tuke Centre and Naomi Unit), and 23 were recruited as outpatients *via* the *Beat* website, which is the UK’s leading charity supporting those suffering with EDs. Specifically, the study was advertised *via* the *Beat* research page and promoted *via* the charity’s email distribution list. Inclusion criterion for the ED group was a clinical diagnosis of an ED, with no restrictions on previous ED diagnosis. Participants recruited *via* The Retreat had a clinical diagnosis confirmed by the patients’ psychiatrist, with participants recruited *via* *Beat* providing a self-reported ED diagnosis, with subsequent assessment from all participants using Eating Disorder Examination Questionnaire (EDE-Q). Such recruitment of clinical individuals *via* self-reported diagnosis has been used in previous research (61). Thirty-one female HCs [mean age, 19.10 (SD, ± 1.27) years] were recruited *via* the University of York, who participated in the present study in return for course credit. Inclusion criteria for the HC group were no current or previous neurological/psychological disorders (self-report). In addition, HCs were explicitly screened for the presence of an ED using an established clinical cutoff of a global EDE-Q score greater than 2.8 (62). All participants were required to be older than 18 years, with no physical condition on their arm or hand that would prevent them from performing the experiment (e.g. severe eczema, scarring, psoriasis). Two ED participants (1 × AN diagnosis, 1 × BN diagnosis) whose age was ≥2 SDs above the group mean (64 and 60 years) were excluded from data analysis. Seven HC participants were excluded from data analysis; one self-reported a current psychological disorder, and six had a global EDE-Q score above the 2.8 global clinical cutoff. Therefore, the final sample size for analysis was 26 ED participants and 24 HC participants. Participant demographic information for both groups following exclusion can be seen in **Table 1**.

**Table 1:** Participant Demographic Information—Means (Standard Deviations) for ED group and HC group.

	ED Group (*N* = 26)	HC Group (*N* = 24)	*t*	*p*	Cohen’s *d*
**Age**	23.46 (5.95)	19.13 (1.42)	3.60	.001	1.00
**BMI**	19.80 (4.39)^a^	20.75 (2.30)	-.96	.344	.27
**Illness duration (years)**	6.39 (5.56)	–	–	–	–
**Treatment duration (years)**	2.73 (2.22)	–	–	–	–
**Restraint** **^b^**	3.70 (2.40–4.80)	1.00 (.40–1.80)	−5.12	<.001^c^	.72
**Eating concern** **^b^**	3.80 (3.00–4.60)	.60 (.60–1.15)	−5.76	<.001^c^	.81
**Shape concern** **^b^**	5.19 (4.23–5.75)	2.35 (.93–3.10)	−5.43	<.001^c^	.77
**Weight concern** **^b^**	4.60 (3.50–5.40)	1.50 (.50–2.75)	−5.49	<.001^c^	.78
**EDE-Q Global** **^b^**	4.21 (3.47–4.94)	1.55 (.69–2.23)	−5.83	<.001^c^	.82

p values corrected for multiple comparisons using false discovery rate (64)*.*

BMI, body mass index.

a ED Group (N = 25). ^b^Median of EDE-Q subscale and global scores with interquartile range in parentheses. ^c^Mann-Whitney U statistic with r value effect size.

### Materials

**Figure 1:**
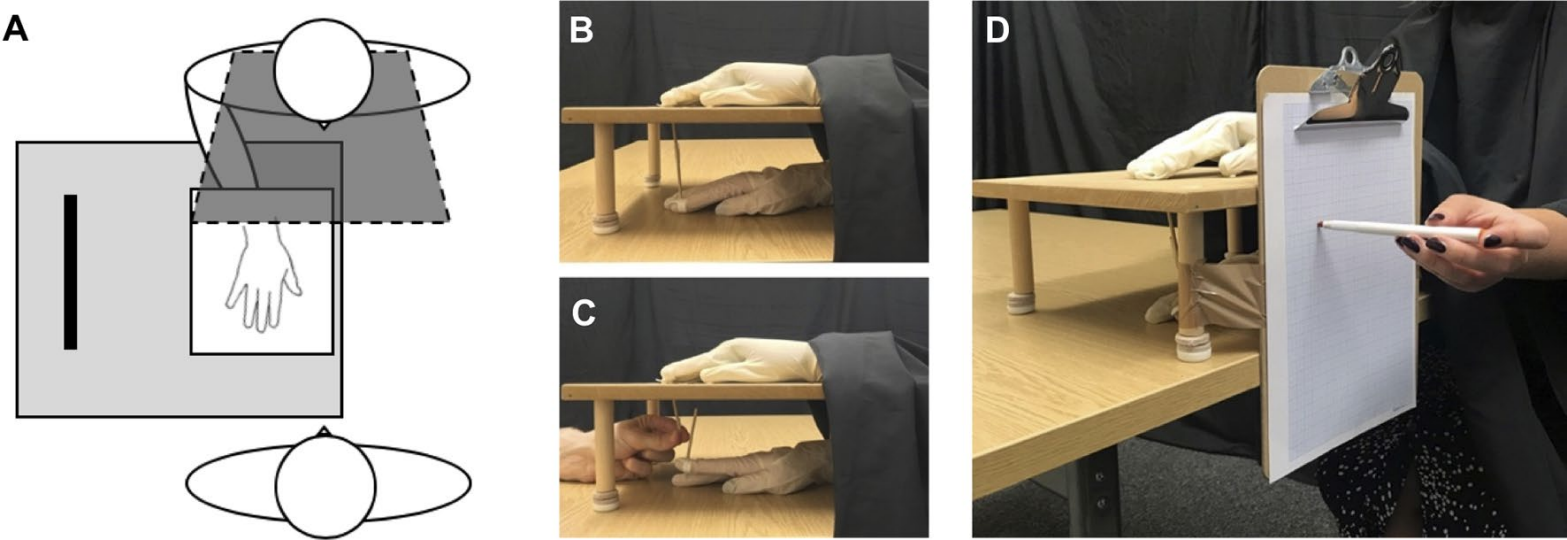
Experimental set-up. **(A)** Participants sat opposite the experimenter and placed their right hand under the platform, directly below the fake hand, which was viewed on top, with a black cape covering their right arm. For each measure within the illusion, participants completed synchronous **(B)** conditions, in which the connection between the two hands was attached, and asynchronous **(C)** conditions, in which the connection was detached, and the experimenter moved the fake hand independently from participant’s own hand. **(D)** Proprioceptive drift measure, in which participants closed their eyes and indicated the felt location of their right index finger, using a coloured marker pen on the grid paper attached to the side of the set-up.

Experimental materials involved a wooden platform (35 × 30 × 13 cm; **Figure 1**) positioned on a table, on top of which was resting a life-sized wooden artist’s right hand (measuring 30 cm from base of the wrist to tip of the middle finger), wearing a latex glove with the palm faced down. Participants were seated at the table and asked to wear an identical latex glove on their right hand, which they then placed underneath the wooden platform, directly below the model hand (**Figure 1**). The participant’s left hand was in a resting position and kept still by their side. Participants wore a black cape around their neck, which occluded their right forearm and the open wrist of the fake hand on the wooden platform, to appear in an anatomically congruent position to the fake hand. A plastic finger cap was then placed on the tip of participant’s right index finger, which was mechanically connected to a matching finger cap on the fake hand by a thin wooden dowel passing through a small hole in the wooden platform, which was attached/detached for the respective experimental condition (see *Procedure* section). Experimental trials and responses for both the mRHI and IAT were made using PsychoPy 2 (63) on an Apple iMac computer (1.6 GHz dual-core Intel Core i5 processor).

### Measures

#### Moving Rubber Hand Illusion

##### Questionnaire

Following experimental trials, the subjective experience of the illusion was recorded using a 12-statement illusion questionnaire (**Table 2**), adapted from previous studies (37). This questionnaire was composed of two subcomponents, addressing the feeling of ownership towards the fake hand (three items) and feeling of agency over the movements of the fake hand (three items). A further six control statements (three ownership control, three agency control) served to control for participant compliance and suggestibility. Participants were asked to rate the extent to which they agreed with each statement on a seven-point Likert scale (−3 strongly disagree to +3 strongly agree) specifically based on the previous trial. All statements were presented in a randomised order.

**Table 2:** Questionnaire for the moving rubber hand illusion, comprising 12 statements that participants rated on a seven-point Likert scale (−3 strongly disagree to +3 strongly agree).

Questionnaire Statement	Category
1. I felt as if I was looking at my own hand. 2. I felt as if the rubber hand was part of my body. 3. I felt as if the rubber hand was my hand.	**Ownership**
4. I felt as if my real hand were turning rubbery. 5. It seems as if I had more than one right hand. 6. It felt as if I had no longer a right hand, as if my right hand had disappeared.	**Ownership Control**
7. The rubber hand moved just like I wanted it to, as if it was obeying my will. 8. I felt as if I was controlling the movements of the rubber hand. 9. I felt as if I was causing the movement I saw.	**Agency**
10. I felt as if the rubber hand was controlling my will. 11. I felt as if the rubber hand were controlling me. 12. It seemed as if the rubber hand had a will of its own.	**Agency Control**

##### Proprioceptive Drift

With eyes closed, participants estimated the perceived height of their unseen, right index finger using an A4 sheet of (millimeter grid) graph paper attached to the side of the experimental set-up (**Figure 1D**). Participants were required to make one swift, but accurate pointing movement towards the graph paper using a coloured marker pen held in their left hand. Each pointing movement was completed three times, with the starting point randomised between participants’ nose, shoulder, or hip, to account for learned motor movement. An average pointing estimation was calculated across the three responses, with pointing movements measured both pre-experimental and postexperimental trials.

##### Hand Size Estimation

Participants were asked to estimate the width of their own hand (at the widest point) prior to the illusion (baseline estimation) and post-experimental trial (13). Both the fake hand and the participants’ own hand were hidden from view using an occluding box during all hand size estimations. For each estimation, the experimenter moved two pointers of a calliper alongside the back of the set-up, occluding their own hands to prevent any further visual cues. Estimations were made with the two pointers of the calliper, once moving towards each other (inwards) and once with pointers moving away from each other (outwards). Participants made their judgements by verbally indicating the point at which their hand would fit precisely between the two pointers. The order of calliper movement (inwards/outwards) was counterbalanced across all participants. A baseline estimation was first made before the illusion, with subsequent post-experimental estimations made following each trial. Changes in hand size estimation were calculated by subtracting the average width of post-trial estimations from the baseline estimation. Participants’ actual hand size was measured at the end of the experiment.

#### Body Satisfaction

##### Explicit Body Satisfaction

A continuous visual analogue scale (VAS), ranging from 0 to 100, was used to assess participant’s explicit, state body satisfaction. Participants were asked, ‘*Right now, how satisfied do you feel with your body?*’ with the scale anchored by ‘extremely dissatisfied’ (0) and ‘extremely satisfied’ (100) (60, 65). Visual analogue scale items have been shown to have good convergent validity with other measures of body satisfaction (66).

##### Implicit Body Satisfaction

**Figure 2:**
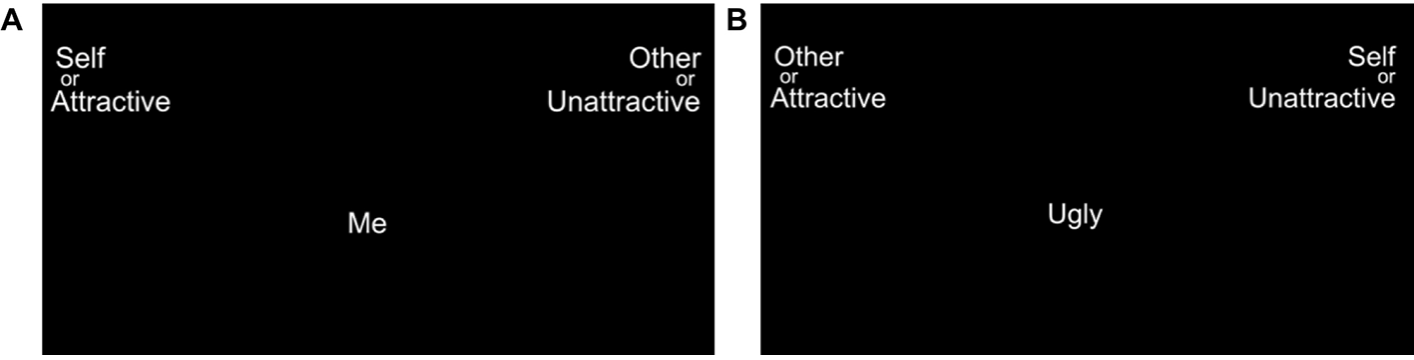
Screenshot depicting example trials within IAT. **(A)** Example compatible condition, in which *Self* and *Attractive* categories (plus *Other* and *Unattractive*) are paired on the same side of the screen. **(B)** Example Incompatible condition in which *Self* and *Unattractive* categories (plus *Other* and *Attractive*) are paired on the same side of the screen. Target words appeared in the centre of the screen, with participants responding by categorising the target words into the left or right of the screen.

Implicit body satisfaction was measured using the IAT (46), in which participants were instructed to categorise target words appearing in the centre of the screen into one of four categories, using only two response options (left/right) (**Figure 2**). Within the body satisfaction IAT [adapted from (52, 53)], target categories were *Self* and *Other*, and attribute categories were *Attractive* and *Unattractive*, with pairings from each category appearing in the top left/right corner of the screen. Target words were chosen based on pilot data from an independent sample, to ensure that words were appropriate and culturally relevant for the present study. Target words and their respective categories can be seen in **Table 3**.

**Table 3:** Implicit Association Test categories and attributes (adapted from 52).

Stimuli Category			
Self	Other	Attractive	Unattractive
Mine	They	Beautiful	Ugly
My	Them	Gorgeous	Unappealing
Me	Their	Good-looking	Bad-looking
Self	Other	Attractive	Unattractive

In the compatible condition, *Self* and *Attractive* categories (plus *Other* and *Unattractive*) were paired on the same side of the screen. In the incompatible condition, the configuration of the categories was switched, in which *Self* and *Unattractive* categories (plus *Other* and *Attractive*) are paired on the same side of the screen (**Figures 2A, B**). The strength of the participants’ implicit cognitions is measured by the difference in the mean reaction times between compatible and incompatible conditions. Faster reaction times indicate that the categorisation of words was more congruent with the individual’s implicit cognitions towards those concepts. Thus, higher body satisfaction equates to stronger associations (i.e. faster reaction times) between compatible condition pairings, compared with incompatible condition parings.

#### Eating Disorder Examination Questionnaire

The EDE-Q is a 28-item questionnaire used as a self-report measure of ED psychopathology (67) amongst clinical and nonclinical populations. The questionnaire assesses disordered eating behaviours within the past 28 days, in which there are four subscales: *Restraint*, *Eating* *Concern*, *Weight* *Concern*, and *Shape* *Concern*. A global score is calculated from the average of the four subscales. Items are rated along a 7-point Likert scale, ranging from ‘0’ to ‘6’, in which higher scores signify higher ED psychopathology. This scoring is with the exemption of six items in which frequency of eating behaviour is recorded; however, these items do not contribute to the subscale scores and were not used in the present study, with ED psychopathology assessed based on the 22-item attitudinal scores. The EDE-Q has been shown to have good internal consistency, with Cronbach’s α ranging from .70 to .83 in a clinical sample (68) and from .78 to .93 in a nonclinical sample (69). In the present study, the overall global EDE-Q measure had a Cronbach’s α of.87 for ED group and .91 for HC group.

### Procedure

#### Moving Rubber Hand Illusion

Participants were first familiarised with the experimental set-up and given instructions of the task procedure. During all conditions, participants sat at the table and placed their right hand underneath the wooden platform, with a plastic finger cap placed on their right index finger. In each trial, the participant’s task was to tap his/her right index finger in a semiregular rhythm for 60 s at approximately one tap per second and was instructed to perform an additional quick ‘double tap’ at random intervals to avoid perfectly regular visuosomatic correlations, which is reported to weaken the illusion (37). Participants were first required to practice the tapping movement prior to experimental trials and were instructed to focus their gaze on the model hand for the duration of each trial.

During synchronous conditions, the mechanical connection (dowel connecting the real and fake index finger) lifted and lowered the right index finger of the fake hand such that movements of the fake hand were in synchrony with the movements of participants’ own right index finger. During asynchronous conditions, the mechanical connection between the real and fake hand was detached, with the movements of the fake index finger controlled by the experimenter moving the dowel with a temporal delay (∼ 500 ms) to participant’s own movements. The experimental procedure consisted of six 60-s trials; three synchronous (illusion) and three asynchronous (control). Each of the three experimental measures (see *Measures* section) was completed once per condition (3 × synchronous, 3 × asynchronous) in separate trials. Condition order was counterbalanced across participants. Between each trial, participants were given a rest period of ∼60 s, in which they removed their right hand from the set-up and flexed their hand/wrist to abolish any carry-over effects.

#### Body Satisfaction

In addition to an explicit measure of state body satisfaction (see *Measures* section), participants’ implicit body satisfaction was measured using the IAT. Participants were first familiarised with the IAT task by completing practice blocks, in which only two categories were presented on the screen (i.e. top left and right of the screen). Participants were instructed to categorise the target words as quickly and accurately as possible using the ‘*Z*’ (left) and the ‘*M*’ (right) key, respectively. Data from practice blocks were not used in any subsequent analysis. In critical (experimental) conditions, each target word belonged to one of four categories, of which two were positioned on the left of the screen and two were positioned on the right (see *Measures* section). All participants completed two experimental blocks of the IAT (1 × Compatible, 1 × Incompatible), each consisting of 120 trials. All target words were presented individually in the centre of the screen, in a randomised order within each block for all participants. The order of conditions and category configurations were counterbalanced across all participants. Following the IAT, participants completed demographic information and the EDE-Q. The duration of the experiment in total was approximately 60 min.

### Data Analysis

Prior to analysis, all data were tested for normality using a Shapiro-Wilk test. When the assumption of normality was not violated (*p* > .05), appropriate parametric tests were used, which are described below. When normality was violated (*p* < .05) or the data were ordinal, nonparametric Wilcoxon signed-ranks tests were used for within-subject analysis and Mann-Whitney *U* tests for between-subject analysis. Nonparametric correlations were analysed using Spearman’s rank correlation. All analyses that directly tested *a priori* hypotheses are uncorrected critical α values, with all other analyses using Bonferroni-corrected α values (stated as necessary below). Effect sizes for parametric tests are indicated by partial η^2^ (η_p_^2^) or Cohen’s *d*, and nonparametric (Wilcoxon signed-ranks and Mann-Whitney *U*) tests are indicated by *r* values, which are equivalent to Cohen’s *d* (70). All statistical analyses were conducted using SPSS version 23.0 (IBM, Chicago, IL, USA).

#### Moving Rubber Hand Illusion

For the subjective measures of ownership and agency (and respective control scores) from the questionnaire ratings, scores were calculated by averaging the individual statements within their respective categories (**Table 2**) to obtain a single score per subscale for each participant (37, 71). First, ownership and agency ratings were compared with their respective control subscale ratings to determine the reliability of the illusion scores in each group, as control scores are not expected to score highly, irrespective of illusion conditions. Control scores are particularly important when testing patient populations, to ensure that participants are not simply complying with all trials and providing high ratings to all questionnaire items (13). Second, ownership and agency scores were compared between synchronous (illusion) and asynchronous (control) conditions to determine the effect of visuomotor synchrony towards subjective illusory experience. Third, ownership and agency scores were independently compared between the ED group and HC group to directly test the hypothesis that ED individuals would show greater instability in their subjective experience body ownership and sense of agency towards the fake hand, following the illusion.

Proprioceptive drift was calculated by subtracting the average height of the pretrial estimation from the posttrial estimation within the pointing task. Positive values signify an upwards drift in the participants’ perceived hand position, and thus an increased illusory experience (25, 37). For hand size estimation measures, the hand width of the fake hand was first compared with the participant’s actual hand size for each group, with actual hand size subsequently compared between the ED and HC groups. Moreover, to test the hypothesis that ED individuals would display an initial overestimation of hand size prior to the illusion compared with HCs, actual hand size was compared with the participant’s baseline estimation of hand width within each group. Next, to investigate whether the effects of the illusion led to a decrease in hand size estimations, difference scores were calculated by subtracting postexperimental estimations from baseline estimations for each participant, per condition. Thus, positive values would signify a decrease in hand size estimation following experimental trials.

#### Body Satisfaction

Explicit ratings of state body satisfaction taken from VAS scores were compared between the ED and HC groups to test our prediction that ED individuals would display a significantly lower explicit body satisfaction. Additionally, the IAT was used as a proxy for implicit body satisfaction. In line with previous research (46), the first two trials of each condition block with the IAT were removed along with all incorrect trials and reaction times outside of lower (300 ms) and upper (3000 ms) boundaries. Data were transformed using a *D* score algorithm, which was calculated as the difference in mean reaction times between compatible and incompatible trials, divided by the inclusive SD across both conditions (72). To directly test the hypothesis that ED individuals would display a significantly lower implicit body satisfaction, *D* scores were compared between the ED and HC groups. In addition, mean reaction times were analysed *via* a 2 × 2 mixed-effects analysis of variance (ANOVA) to investigate whether any group differences are driven by the compatibility of the trials, in which slower reaction times within compatible trials would signify a reduced implicit self-serving body image bias. Thus, compatibility (compatible vs. incompatible) was entered as the within-subjects factor, and group (ED group vs. HC group) entered as the between-subjects factor.

#### Correlational Analyses

To directly investigate the hypothesis that perceptual and cognitive-affective components of body image would relate with each other, the association between the above measures within the mRHI and body satisfaction tasks was explored using a nonparametric Spearman’s rank correlation. Moreover, correlations were also explored between perceptual and cognitive-affective measures with ED psychopathology, using the EDE-Q.

## Results

### Moving Rubber Hand Illusion

#### Questionnaire

**Figure 3:**
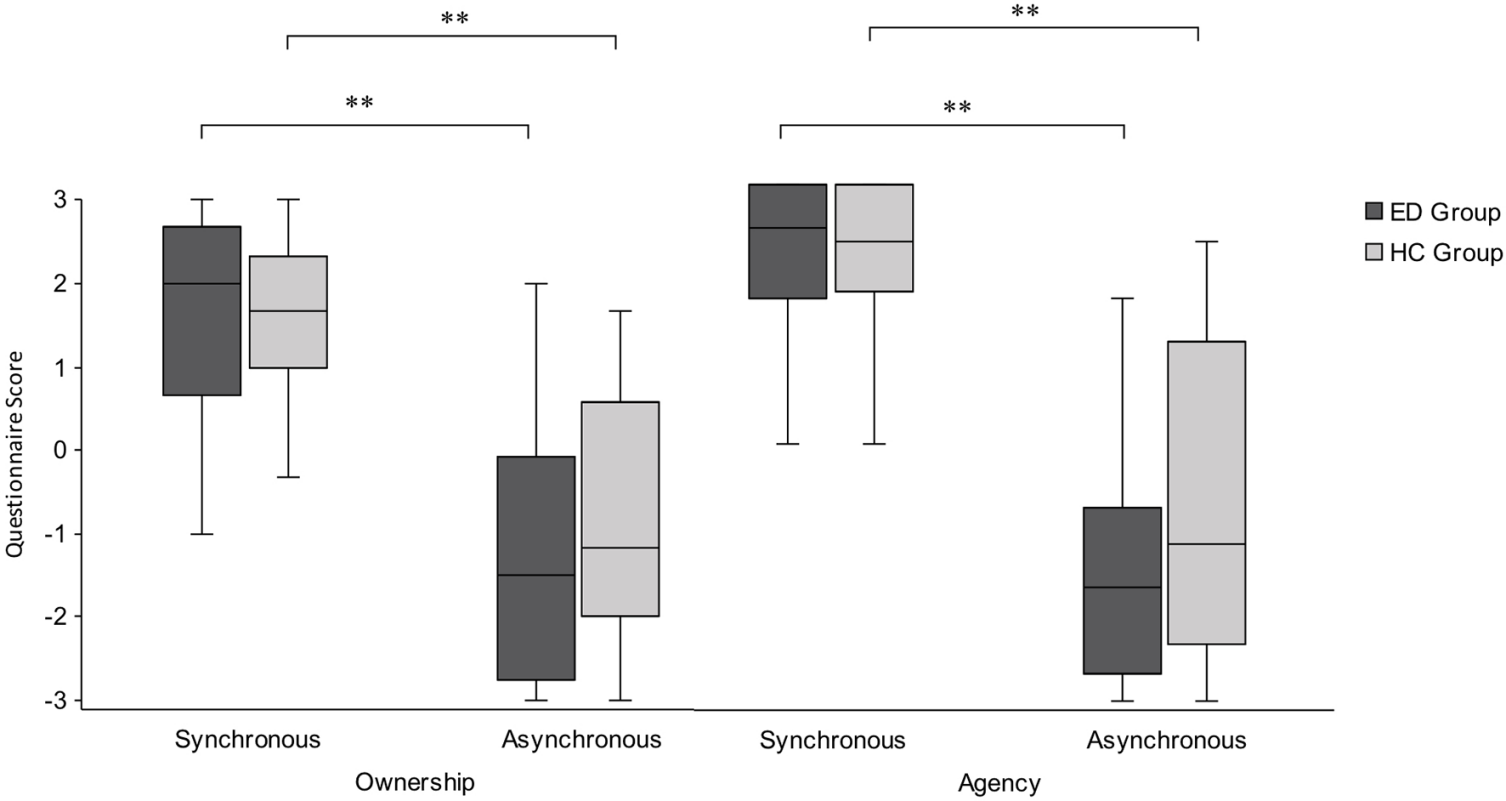
Boxplot displaying ownership and agency scores from the mRHI questionnaire, presented by condition and group. Significantly greater subjective ownership and agency were observed following synchronous compared with asynchronous conditions, with no significant difference in subjective ownership or agency between the ED and HC groups. Intersecting line = median; box = upper and lower interquartile range; whiskers = minimum and maximum values. **p < .001.

Data from subscales within the mRHI questionnaire were ordinal and found to be non-normal in the majority of cases (Shapiro-Wilk *p* < .05); therefore, appropriate nonparametric tests were used. First, a Wilcoxon signed-rank test revealed that illusory ownership was induced for both the ED group (*Z* = −4.03, *p* < .001, *r* = 0.79) and HC group (*Z* = −3.88, *p* < .001, *r* = 0.79), with significantly higher scores in response to ownership questions compared with ownership control questions, following synchronous conditions. Next, a further Wilcoxon signed-ranks test revealed a significant effect of synchrony for both the ED group (*Z* = −4.29, *p* < .001, *r* = 0.84) and HC group (*Z* = −4.29, *p* < .001, *r* = 0.88), with higher ownership scores following synchronous compared with asynchronous conditions (**Figure 3**). Finally, a Mann-Whitney *U* test revealed no significant difference between groups following synchronous conditions (*U* = 300.00, *Z* = −0.24, *p* = .815, *r* = 0.03) or asynchronous conditions (*U* = 283.00, *Z* = −0.57, *p* = .572, *r* = 0.08).

The same analyses were conducted for agency scores, in which a Wilcoxon signed-ranks test revealed that illusory agency was induced for both the ED group (*Z* = −4.46, *p* < .001 *r* = 0.87) and HC group (*Z* = −4.22, *p* < .001, *r* = 0.86), with significantly higher scores in response to agency questions compared with agency control questions, following synchronous conditions. Next, a further Wilcoxon signed-ranks test revealed a significant effect of synchrony for both ED group (*Z* = −4.29, *p* < .001, *r* = 0.84) and HC group (*Z* = −4.20, *p* < .001, *r* = 0.86), with higher agency scores following synchronous compared with asynchronous conditions (**Figure 3**). Finally, a Mann-Whitney *U* test revealed no significant difference between groups following synchronous conditions (*U* = 290.50, *Z* = −0.43, *p* = .668, *r* = 0.06) or asynchronous conditions (*U* = 259.00, *Z* = −1.03, *p* = .301, *r* = 0.15). Taken together, these results suggest that ED and HC groups show a significantly stronger illusory experience following synchronous conditions compared with asynchronous conditions, but had an equally strong subjective experience of ownership and agency towards the fake hand.

#### Proprioceptive Drift

Following synchronous conditions, mean proprioceptive drift was 7.68 (SD, ± 24.80) mm for the ED group and 9.67 (SD, ± 17.05) mm for the HC group. Following asynchronous conditions, mean proprioceptive drift was 5.62 (SD, ± 17.05) mm for the ED group and−0.85 mm (SD, ± 22.90) mm for the HC group. As proprioceptive drift data were normally distributed for both groups (Shapiro-Wilk *p* > .05), a parametric 2 × 2 mixed-effects ANOVA was run, with visuomotor synchrony (synchronous vs. asynchronous) as the within-subjects factor and group (ED group vs. HC group) as the between-subjects factor. In contrast with previous research, no main effect of visuomotor synchrony was observed between synchronous and asynchronous conditions (*F* (1,48) = 2.66, *p* = .109, η_p_^2^ = 0.05). Moreover, no significant main effect of group was observed (*F*_1,48_ = 0.27, *p* = .604, η_p_^2^ =^ .^01), and no interaction between visuomotor synchrony and group was observed (*F*_1,48_ = 1.21, *p* = .277, η_p_^2^ = 0.03).

#### Hand Size Estimation

Hand size estimation data were normally distributed across the whole sample (Shapiro-Wilk *p* > .05); therefore, appropriate parametric tests were used. First, an independent-samples *t* test revealed that there was no significant difference in actual hand width (in millimetres) between the ED group and the HC group (**Table 4**) (*t*_48_ = −0.295, *p* = .77, *d* = 0.08). Second, paired-samples *t* tests revealed that the width of the fake hand (74 mm) was significantly narrower compared with the actual hand width of the ED group (*t*_25_ = −2.89, *p* = .008, *d* = 0.57) and the HC group (*t*_23_ = −3.26, *p* = .003, *d* = 0.67). Finally, to directly test the hypothesis that ED individuals would overestimate their hand size prior to the illusion, actual hand size was compared with participants’ baseline estimation of hand width for each group (**Table 4**) using paired-samples *t* tests. Participants in the ED group significantly overestimated their own hand width, prior to the illusion (*t*_25_ = −3.33,* p* = .003, *d* = 0.65). Additionally, participants in the HC group also significantly overestimated their own hand width, prior to the illusion (*t*_23_ = −2.15, *p* = .043, *d* = 0.44). Hand size overestimations did not significantly differ between the ED and HC groups (*t*_48_ = 0.76, *p* = −.453, *d* = 0.21).

**Table 4:** Hand size dimensions and estimations. Actual hand size dimensions (mean and SD) of participants with baseline estimations and post-experimental estimations. Units measured in millimeters (mm).

	ED Group (*N* = 26)	HC Group (*N* = 24)	*t*	*p*	Cohen’s *d*
**Actual Hand Width**	76.00 (3.53)	76.29 (3.44)	−.295	.769	.08
**Baseline Estimation**	83.90 (12.97)	81.60 (11.10)	.675	.506	.19
**Synchronous – Post-experimental Estimation**	78.79 (12.20)	78.33 (6.87)	.164	.870	.05
**Asynchronous – Post-experimental Estimation**	79.71 (11.44)	80.00 (9.48)	−.097	.923	.03

Next, to directly test the hypothesis that ED individuals would report a significant decrease in hand size estimation after the illusion was induced, difference scores were calculated for each group by subtracting post-experimental estimations from the baseline estimation. Difference scores were compared to zero *via* a one-sample *t* test, in which positive values would indicate a decrease in hand size estimation following the illusion. For the ED group, participants reported significantly lower hand size estimations following induction of the illusion, for both synchronous conditions (*t*_25_ = 2.84, *p* = .009, *d* = 0.56) and asynchronous conditions (*t*_25_ = 2.74, *p* = .011, *d* = 0.54). Interestingly, for the HC group, participants also reported a significantly lower hand size estimation following induction of the illusion for synchronous conditions (*t*_23_ = 2.09, *p* = .048, *d* = 0.43), but not for asynchronous conditions (*t*_23_ = 1.10, *p* = .281, *d* = 0.22) (**Table 4**).

Finally, postexperimental hand size estimations were compared with participant’s actual hand size, to determine whether such estimations reflected a more veridical measurement of hand width. For the ED group, paired-samples *t* tests revealed no significant differences between actual hand size and postexperimental estimations following synchronous (*t*_25_ = −1.15, *p* = .259, *d* = 0.23) or asynchronous conditions (*t*_25_ = −1.68, *p* = .106,* d* = 0.33). Crucially, baseline estimations made prior to the illusion were significantly different from actual hand size; therefore, this nonsignificant result reflects a reduction in hand size estimation, which is closer to the ED participant’s actual hand size. Similarly, for the HC group, paired-samples *t* tests revealed no significant differences between actual hand size and postexperimental estimations following synchronous (*t*_23_ = −1.29, *p* = .208, *d* = 0.26) or asynchronous conditions (*t*_23_ = −1.82, *p* = .082, *d* = 0.37). Taken together, the above results suggest that the ED group showed a significant reduction in hand size estimation following induction of the illusion following both synchronous and asynchronous conditions, which is closer to their veridical hand size. Whilst the HC group also displayed a more accurate estimation of their hand width following synchronous conditions, this was not matched following asynchronous conditions. Moreover, difference scores in the ED group were more pronounced as shown by a larger effect size, which may reflect a greater malleability of body representation within this group.

### Body Satisfaction

#### Explicit Body Satisfaction

Data from the VAS ratings were non–normally distributed across the whole sample (Shapiro-Wilk *p* < .05); therefore, a nonparametric Mann-Whitney *U* test was used to compare state body satisfaction between the ED group and HC group. As predicted, the ED group reported a significantly lower state body satisfaction (median, 15.00) compared with the HC group (median, 63.00; *U* = 33.00, *Z* = −5.42, *p* < .001).

#### Implicit Body Satisfaction

**Figure 4:**
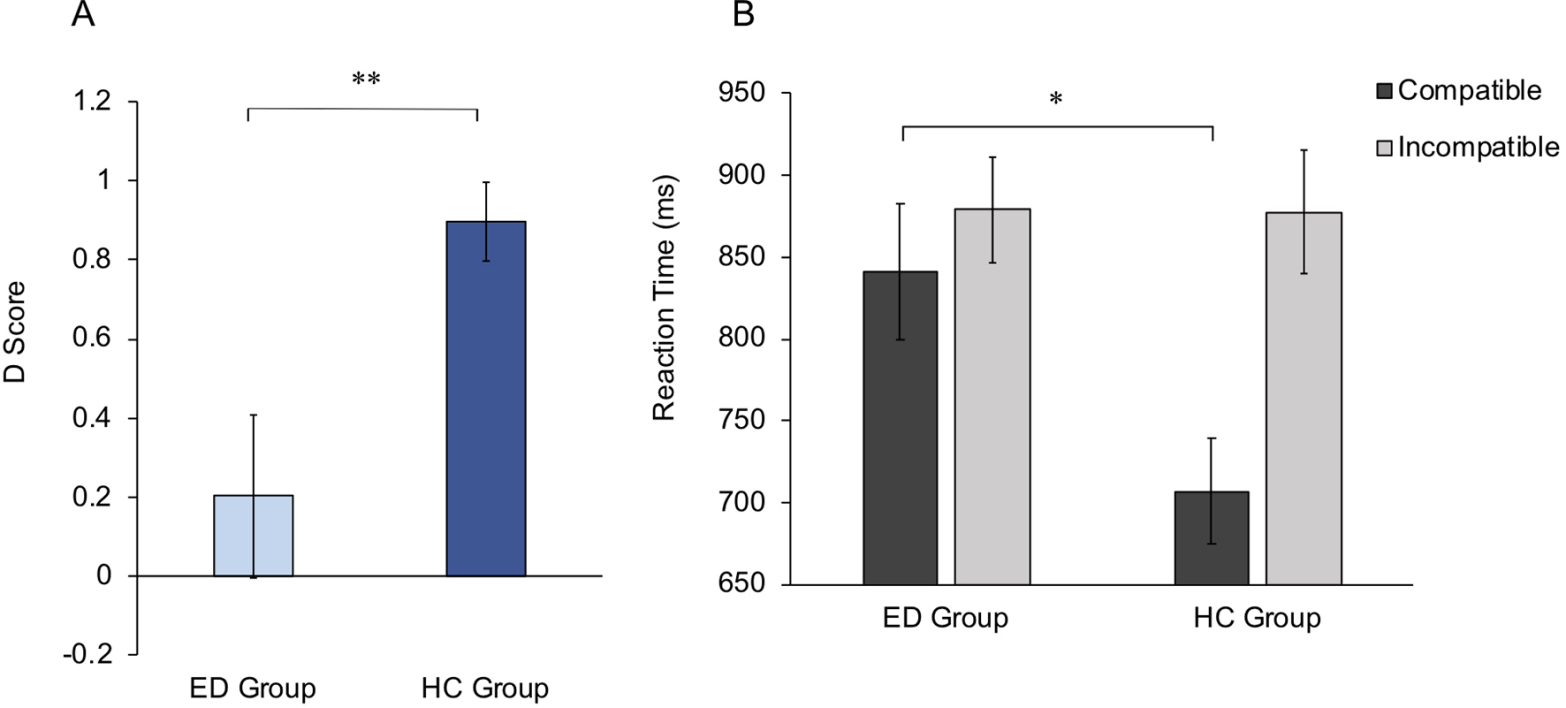
Implicit Association Test scores. **(A)** Mean *D* scores for ED and HC groups. Higher *D* scores indicate higher implicit body satisfaction within the HC group compared with the ED group. **(B)** Mean reaction times for Compatible and Incompatible trials, for ED and HC groups. Group differences are shown to be driven by significantly slower reaction times in the ED group compared with the HC group, within Compatible trials. Error bars for both graphs show standard error. *p < .05, **p < .01.

To directly test the hypothesis that the ED group would display lower implicit body satisfaction compared with the HC group, *D* scores from the IAT were compared between groups. Note that lower *D* scores represent lower implicit body satisfaction. Data from the IAT were normally distributed (Shapiro-Wilk *p* > .05); therefore, an independent-samples *t* test was run, which revealed a significantly lower *D* score within the ED group (mean, 0.20) compared with the HC group (mean, 0.90; *t*_35.86_ = −3.06, *p* = .004, *d* = 0.43). This suggests that ED participants displayed a reduced body satisfaction on an implicit level compared with HCs. *D* scores for both groups are shown in **Figure 4A**.

To further investigate whether ED individuals show a reduced implicit self-serving body image bias within the IAT, mean reaction times for each condition were entered into a 2 × 2 mixed-effects ANOVA, with condition (Compatible vs. Incompatible) as the within-subjects factor and group (ED group vs. HC group) as the between-subjects factor. A main effect of condition was observed (*F*_1,48_ = 22.43, *p* < .001, η_p_^2^ = 0.32), with significantly lower reaction times following Compatible versus Incompatible conditions. No main effect of group was observed (*F*_1,48_ = 2.15, *p* = .149, η_p_^2^ = 0.04). However, a significant interaction was observed between condition and group (*F*_1,48_ = 9.00, *p* = .004, η_p_^2^ = 0.16). Thus, Bonferroni-corrected independent-samples *t* tests (critical α = .025) revealed a significant difference between groups following Compatible (*t*_48_ = 2.52, *p* = .015, *d* = 0.36), but not Incompatible conditions (*t*_48_ = 0.04, *p* = .972, *d* = 0.01) (**Figure 4B**). This suggests that differences in implicit attitudes between the ED and HC groups are driven specifically by weaker associations between attractiveness and the self within ED individuals.

#### Relationship Between Explicit and Implicit Body Satisfaction

To investigate whether explicit measures of body satisfaction related to performance on the IAT, a correlation analysis was run across the whole sample (N = 50). A Spearman’s rank correlation revealed a significant positive correlation between state body satisfaction and *D* scores on the IAT across the whole sample (*r* = 0.46, *p* = .001), which may suggest that those with higher explicit body satisfaction also display a higher implicit body satisfaction. Furthermore, Bonferroni-corrected Spearman’s rank correlations (critical α = .025) revealed that lower state body satisfaction is driven by performance on Compatible trials (i.e. *Self* and *Attractive* categories paired) within the IAT, with a significant negative correlation between state body satisfaction and Compatible trials (*r* = −0.34, *p* = .014) but not with Incompatible trials (*r* = 0.10, *p* = .485).

### Correlational Analyses

To directly test the hypothesis that perceptual and cognitive-affective components of body image would relate with each other, measures from the mRHI (questionnaire scores, proprioceptive drift, hand size estimation) were correlated with body satisfaction measures (explicit and implicit) across the whole sample. A Spearman’s rank correlation revealed a significant positive correlation between synchronous ownership questionnaire scores and IAT *D* scores (*r* = 0.32, *p* = .022), which was driven by the ED group scores (see **Supplementary Materials** for full tables). Moreover, a significant positive correlation was observed between synchronous proprioceptive drift scores and IAT *D* scores (*r* = 0.30, *p* = .032), which was similarly driven by scores in the ED group. This suggests that a stronger explicit and implicit experience of the illusion is associated with increased implicit body satisfaction, which highlights that a link does exist between perceptual and cognitive-affective components of body image. No further noteworthy correlations were observed between the above measures (see **Supplementary Materials** for full tables).

Finally, to investigate the relationship between body perception and body satisfaction with ED psychopathology, the above measures were correlated with scores from the EDE-Q across the whole sample. A Spearman’s rank correlation revealed no noteworthy correlations between perceptual measures on the mRHI and EDE-Q scores across the whole sample (see **Supplementary Materials** for full tables). However, as expected, a significant negative relationship was observed between EDE-Q global scores and explicit body satisfaction (*r* = −0.794, *p* < .001), showing that those with higher ED psychopathology reported lower state body satisfaction. Interestingly, a significant negative relationship was also observed between EDE-Q global scores and *D* scores within the IAT (*r* = −0.35, *p* = .012), which suggests that those with higher ED psychopathology also display a lower implicit body satisfaction. This relationship is shown to be specifically driven by subscale scores relating to *Shape Concern* (*r* = −0.47, *p* = .001) and *Weight Concern* (*r* = −0.41, *p* = .003), which reflect body-related attitudes, rather than attitudes towards eating behaviours (i.e. *Restraint/Eating Concern*), which showed no significant relationship with IAT *D* scores (see **Supplementary Materials** for full tables).

## Discussion

The present study investigated the perceptual and cognitive-affective components of body image within ED individuals and healthy females. Specifically, the multisensory mRHI was used to assess body ownership and agency, alongside explicit and implicit measures of body satisfaction. Following induction to the illusion, results showed that both ED and HC individuals displayed a similar subjective experience of illusory ownership and agency towards the fake hand. Moreover, both groups initially overestimated their own hand width prior to the illusion, with a significant reduction in overestimation in ED group following both synchronous and asynchronous conditions, which was not mirrored to the same degree in the HC group. Second, ED individuals displayed significantly lower satisfaction towards their body compared with healthy females, on both an explicit and implicit level. Such implicit findings were shown to be driven specifically by a weaker association between words relating to the self and attractiveness. Finally, a significant relationship was observed between specific perceptual measures and implicit body satisfaction, which underlines the key link between body perception and body emotion. Taken together, the present findings support previous research by indicating that ED individuals have a more malleable experience of the bodily self, compared with healthy females. Moreover, novel findings show that ED individuals present with a lower implicit satisfaction towards their body that relates with perceptual experience, which may provide important implications within clinical treatment.

Using the mRHI, the present study builds upon previous multisensory integration research within ED groups (13, 24), as being the first to investigate the sense of agency and its interaction with body ownership within this population. Whilst the ‘classic’ RHI incorporates a three-way interaction between visual, tactile, and proprioceptive input (25), the present paradigm is supplemented by efferent, kinaesthetic information from voluntary motor actions, which elicits a sense of body ownership and agency towards a fake hand, both of which are key perceptual components within the bodily self (38). Results showed that both ED and HC groups displayed a strong sense of ownership and agency towards the fake hand following synchronous illusion conditions. However, contrary to hypotheses, the two groups displayed a comparable subjective experience of ownership and agency during the task. This finding is in contrast to previous work that has investigated subjective body ownership within the ‘classic’ RHI, in which ED groups displayed higher sense of ownership towards the fake hand compared with HCs (13, 24). Together, the above results suggest that the subjective sense of ownership and agency may be similar between ED and healthy groups when incorporating voluntary movement towards body representation.

Similarly, despite previous research observing differences in proprioceptive drift between the ED and HC groups (24), the present study is in line with later work that did not observe such effects between groups (13). Many researchers have widely accepted that subjective measures of embodiment following multisensory integration are dissociable from a perceived change in spatial location, which leads to proprioceptive drift (73, 74). However, the observed lack of difference between groups and indeed lack of proprioceptive drift observed from the illusion may be accounted for by a task-dependency effect. Within the present study, participants were asked to make a motor response towards the perceived location of their hand. However, previous research in healthy individuals has suggested a dissociation between perceptual body judgements and motor responses, in which participants showed susceptibility to the ‘classic’ RHI when making a perceptual response (i.e. verbal judgement of hand location) but showed intact proprioceptive judgement when making a motor response (i.e. a pointing movement towards hand location) (75). This suggests that the two measures denote separate body representations; therefore, future research should investigate whether such proprioceptive measures of the mRHI differ between ED and healthy groups when using perceptual, verbal responses of perceived hand location.

The present study provides a valuable foundation to further study the sense of agency within EDs, which remains a largely underresearched topic within this clinical population. Given their close association in contributing towards a coherent body representation (76), it is difficult to dissociate feelings of agency and feelings of ownership within voluntary movement, not least when sensory feedback of movement is likely to further enhance ownership (77). Within the present study, the contribution of sense of agency towards the sense of ownership — and vice versa — cannot be disentangled. Indeed, the observed lack of difference between HC and ED groups in ownership and agency may be accounted for by the enhancement of subjective ownership as a result of subjective agency following synchronous conditions within the mRHI. Thus, previous research that has observed greater plasticity in body ownership amongst ED patients within the ‘classic’ RHI (13, 24) may not be directly comparable to the present study, given the additional, interlinked component of agency influencing such subjective ownership. One method to overcome this in future research would be to first undertake the ‘classic’ RHI to determine the stability of ownership between the ED and HC groups from visuotactile integration (13), before then measuring the stability of body agency when introducing voluntary movement *via* the mRHI. Indeed, research using the mRHI has independently investigated the factors that are known to influence the sense of ownership and agency, in healthy individuals (37, 71, 78) and clinical groups (79). Specifically, anatomical plausibility of the hand and mode of movement has been manipulated, comparing active movement with passive movement (in which the experimenter moves the wooden connection, thus moving the fake hand and participant’s hand). Importantly, such manipulations have been shown to dissociate the sense of agency from the sense of ownership (38). Within the present study, the total number of trials within the illusion task was intentionally limited in order to reduce extensive fatigue for ED groups; therefore, body ownership and agency were not independently manipulated.

Furthermore, results showed that whilst both ED and HC groups displayed an initial overestimation of hand width prior to the illusion, ED individuals displayed a significant reduction in their hand width estimation following both synchronous (illusion) and asynchronous (control) conditions, which was not directly mirrored in healthy females. This finding is in line with previous research (13, 26), suggesting that such perceptual changes from ED individuals occurred irrespective of the subjective experience of the illusion, which was shown to significantly differ between conditions. As previously discussed, research has suggested that greater perceptual effects within multisensory illusions amongst ED populations are associated with an increased malleability of the bodily self, in which such individuals often display a visual dominance that overrides proprioceptive input during the illusion (13, 24, 26). Therefore, an increased sensory weighting towards visual input of the fake hand may have been sufficient to change size estimations of one’s own hand amongst ED individuals, irrespective of the condition. Importantly, the present results support previous research that highlights an inherent instability of perceptual body representation in ED individuals. Such findings have important clinical implications within the treatment of body image disturbance in EDs, by showing that perceptual estimation of body size can be improved within this population (80). Thus, whilst the long-term effects of improved perceptual accuracy of body size remain unknown in ED patients, a more veridical representation of one’s own body is likely to positively impact upon clinical outcomes and the cognitive-affective component of body image disturbance (39, 81).

It must be noted that healthy females did also initially overestimate their hand size prior to the illusion and show a subsequent reduced hand size estimation — but following synchronous conditions only. In other words, healthy females were shown to improve their hand size estimation as a consequence of illusion conditions, which reinforces the effect of multisensory integration in inducing perceptual changes in perceived body size amongst healthy individuals (59). Importantly, the effect was different to the ED group who recorded a reduced estimation following both synchronous and asynchronous conditions, which reinforces the greater malleability of the bodily self in ED individuals compared with HCs. However, it is speculated that initial overestimation from the HC group — which occurred contrary to hypotheses — may be a consequence of higher ED psychopathology within the nonclinical range amongst the present sample. Whilst global EDE-Q scores within the HC group (median, 1.55) were below the clinical cutoff (2.80; 59), such scores appear higher than other European countries that use the EDE-Q in nonclinical samples (e.g. median = .42; 53). Indeed, six HC participants were excluded from the present study after scoring above the clinical cutoff for an EDE-Q global score. Therefore, in addition to the hand size estimation results above, such EDE-Q scores may also, in some part, explain the nonsignificant effects between the ED group and HC group on measures of subjective ownership and agency towards the fake hand. Taken together, such inflated scores amongst a healthy female sample reinforce the need to investigate ED psychopathology and vulnerability in the nonclinical population, and highlight how that EDE-Q may require assessment as a clinical measure within the UK, with regard to normative scores between nonclinical and clinical samples (82).

As shown above (i.e. hand size estimation effects), given the consistent findings in the ED literature that have shown perceptual effects of the illusion following both synchronous and asynchronous conditions, it would be informative for participants to undertake subjective and objective measures of embodiment following mere visual observation of the fake hand, with their own hand hidden from view. This would determine the degree of embodiment experienced by participants due to ‘visual capture’ of congruent visuoproprioceptive information alone, as a baseline measure made prior to visuomotor integration from illusory trials (83–85). As previously discussed, experiment duration was minimised for ED individuals within the present study; therefore, a visual capture measure of embodiment was not taken. However, given the apparent increased sensitivity to visual input amongst ED populations, future research should include such conditions that take such ‘baseline’ measures of embodiment following mere visual observation of a fake body (part), to more precisely delineate the role of altered multisensory integration within ED groups. This would be particularly interesting within an RHI set-up, as evidence has shown a greater perceptual malleability when using the RHI compared with a whole body illusion, in relation to ED psychopathology within healthy groups (83) and clinical ED groups (26).

As hypothesised, explicit measures of state body satisfaction revealed significantly lower self-reported scores in ED groups compared with healthy females. However, to the authors’ knowledge, the present study is the first to investigate implicit body satisfaction in an ED sample, using the IAT. Results on the IAT showed that ED individuals displayed a significantly lower implicit body satisfaction compared with healthy females, with such differences driven by weaker associations between the self and attractiveness. These findings support previous research that suggests ED individuals lack a positive self-serving body image ‘bias’ (7), yet builds further by suggesting that dysfunctional attitudes towards one’s self-appearance are more deeply rooted amongst ED individuals, with such implicit cognitions likely to be more resistant to change or modification compared with explicit, self-reported cognitions (43). Such findings can have important clinical implications for recovery and relapse, in assessing the implicit biases that are not influenced by a patient’s compliance or pressure to report improvement in clinical outcomes following treatment (86). Indeed, recovered ED patients who explicitly self-report improvement in attitudes towards weight and shape following treatment may still be at increased risk of relapse if such cognitions are not addressed on an implicit level, which may play an important role in the prognosis of the disorder (8, 43). This is highlighted in the present study, with implicit body satisfaction shown to be associated with ED psychopathology across the whole sample. Specifically, a significant negative correlation was observed between IAT *D* scores and global EDE-Q scores, which was driven by scores on *Shape Concern* and *Weight Concern* EDE-Q subscales, and not from eating-related subscales (i.e. *Restraint*/*Eating Concern*). Importantly, it is unlikely that this significant correlation across the whole sample was driven by group differences on the above measures, given that significant differences were shown across all EDE-Q subscales between groups (**Table 1**). Therefore, such findings reinforce the link between implicit and explicit cognitions regarding body satisfaction within the pathology of EDs and the need to address both constructs within treatment to improve upon clinical outcomes.

Computer-based paradigms such as the IAT can be a cost-effective method used to assess and improve upon dysfunctional implicit cognitions within ED treatment, alongside traditional, explicit measures of clinical interviews and standardized questionnaires (86). Indeed, increasing research is showing that interventions that target such implicit processes may have clinical efficacy in improving cognitions surrounding one’s body satisfaction (8). Furthermore, whilst the present study used appearance-related word associations within the IAT, it would be interesting for future research to dissociate such implicit biases from general cognitive measures such as self-esteem (87). Indeed, a dissociation between shape- or weight-related cognitions and general self-esteem would suggest that altered cognitions within this population may be specific to the body and would provide researchers and clinicians with a clearer focus within which to target treatment (86).

Finally, results revealed a relationship between perceptual and cognitive-affective components of body image across the whole sample, shown by significant positive correlations between ownership questionnaire scores and proprioceptive drift scores from the mRHI, with implicit body satisfaction from IAT *D* scores. This supports the argument that a direct link does exist between body perception and emotion, with such findings shown to be driven more specifically by ED group scores. However, the direction of such relationships was contrary to hypotheses, as it was predicted that ED individuals would display increased ownership — implicated with an instability in the bodily self — which would be associated with reduced body satisfaction. Whilst the explanation for this effect remains unclear, it could be speculated that individuals with a greater instability in their body perception (i.e. ED individuals) may have less negative implicit attitudes towards their own body because they are attaining their idealised, yet unhealthy, ultrathin body. This would be particularly relevant amongst individuals with AN, in which a strong drive for thinness is a key characteristic within such a diagnosis, with increasingly prevalent ‘thinspiration’ media websites positively reinforcing such aberrant weight loss (88, 89). Importantly, such findings highlight the complexity of the relationship between perceptual and cognitive-affective components of body image, in which further research is required to uncover the most salient conditions in which perceptual alterations relate to emotional bodily experience.

Given the present findings highlighting a relationship between perceptual and cognitive-affective components of body image, future research should investigate how this behavioural relationship is represented in the brain. Recent neuroscientific research has significantly increased our understanding of the neural basis of EDs, with several studies highlighting structural and functional correlates of body image disturbance (90). Specifically, alterations in posterior parietal areas, associated with the integration of sensory information, have been implicated with the perceptual component of body image disturbance amongst AN patients (91, 92). Moreover, prefrontal cortex and insula alterations have been implicated with the affective component of body image disturbance. Therefore, following neuroimaging evidence that has highlighted the interaction between perceptual and affective representations amongst healthy individuals (60), future research should investigate the functional connectivity within the brain amongst ED patients, to determine whether alterations in the communication between the above neural regions would link with the prognosis of the disorder.

The above findings must be considered within the context of limitations of the present study. Whilst a large percentage of the ED group presented with a diagnosis of AN (∼70%), the heterogeneity in diagnosis (e.g. BN, BED) and treatment received (e.g. inpatient/outpatient) from ED individuals may have impacted the results within this group. Given the complexity and heterogeneity of clinical populations, this is a typical methodological issue within the ED literature. Indeed, similar research has shown effects of perceptual instability when using an ED group with varied diagnoses (23). However, the sample size within the present study was smaller than previous research that has included varied ED diagnoses; therefore, future research should undertake such work amongst larger, homogeneous samples of independent ED diagnoses.

In conclusion, the present study is one of the first to investigate the independent roles and relationship between perceptual and cognitive-affective components of body image, amongst ED and HC groups. Using a multisensory illusion paradigm that incorporated active, volitional movement, our results support previous research in highlighting the malleability of the perceptual bodily self amongst ED individuals. Second, ED individuals displayed disturbances in their cognitive-affective component of body image, shown by significantly lower body satisfaction on both an explicit and implicit level compared with healthy females, with altered implicit cognitions shown to be specifically driven specifically by weaker associations between the self and attractiveness. Finally, results highlighted an association between the perceptual and cognitive-affective components of body image, yet further research is required to determine the direct effect between these components within both clinical and nonclinical groups. Taken together, such findings can provide important clinical implications in the treatment of body image disturbance, in identifying perceptual alterations amongst this population that are possible to change, and assess more deeply rooted, negative implicit cognitions, which should be targeted alongside typical self-reported measures of recovery in EDs.

## Data Availability Statement

All datasets generated for this study are included in the manuscript/supplementary files.

## Ethics Statement

The studies involving human participants were reviewed and approved by The NHS Health Research Authority (North East – York Research Ethics Committee; Project ID: 199702) The Retreat Mental Health Care Centre, York (Research Governance Committee) Beat Eating Disorders Charity Research Ethics Committee The University of York Departmental Ethics Committee. The patients/participants provided their written informed consent to participate in this study.

## Author Contributions

MC and CP contributed to the conception and design of the experiment. MC collected and analysed the data under the supervision of CP. MC drafted the manuscript, and CP provided critical revisions. All authors approved the manuscript before submission.

## Conflict of Interest

The authors declare that the research was conducted in the absence of any commercial or financial relationships that could be construed as a potential conflict of interest

## References

[1] Stice E Risk and maintenance factors for eating pathology: a meta-analytic review. Psychol Bull (2002) 128(5):825–48. 10.1037/0033-2909.128.5.825 12206196

[2] American Psychiatric Association Diagnostic and statistical manual of mental disorders (DSM-5^®^) (2013). American Psychiatric Pub. 10.1176/appi.books.9780890425596

[3] Cash TF Deagle EA The nature and extent of body-image disturbances in anorexia nervosa and bulimia nervosa: a meta-analysis. Int J Eat Dis (1997) 22(2):107–25. 10.1002/(SICI)1098-108X(199709)22:2<107:AID-EAT1>3.0.CO;2-J 9261648

[4] Gardner RM Brown DL Body size estimation in anorexia nervosa: a brief review of findings from 2003 through 2013. Psychiatry Res (2014) 219(3):407–10. 10.1016/j.psychres.2014.06.029 25023364

[5] Øverås M Kapstad H Brunborg C Landrø NI Lask B Memory versus perception of body size in patients with anorexia nervosa and healthy controls. Eur Eat Disord Rev (2014) 22(2):109–15. 10.1002/erv.2276 24590562

[6] Mai S Gramann K Herbert BM Friederich HC Warschburger P Pollatos O Electrophysiological evidence for an attentional bias in processing body stimuli in bulimia nervosa. Biol Psychol (2015) 108:105–14. 10.1016/j.biopsycho.2015.03.013 25813122

[7] Jansen A Smeets T Martijn C Nederkoorn C I see what you see: the lack of a self-serving body-image bias in eating disorders. Br J Clin Psychol (2006) 45(1):123–35. 10.1348/014466505X50167 16480571

[8] Martijn C. Alleva J. M. Jansen A. Improving body satisfaction: do strategies targeting the automatic system work? Eur Psychol (2015). 20(1), 62. 10.1027/1016-9040/a000206

[9] Urgesi C Multiple perspectives on body image research. European Psychologist (2015), Vol. 20 pp. 1–5. 10.1027/1016-9040/a000223

[10] Alleva JM Sheeran P Webb TL Martijn C Miles E A meta-analytic review of stand-alone interventions to improve body image. Plos One (2015) 10(9):e0139177. 10.1371/journal.pone.0139177 26418470 PMC4587797

[11] Murphy R Straebler S Cooper Z Fairburn CG Cognitive behavioral therapy for eating disorders. Psychiatr Clin North Am (2010) 33(3):611–27. 10.1016/j.psc.2010.04.004 PMC2928448 20599136

[12] Guardia D Lafargue G Thomas P Dodin V Cottencin O Luyat M Anticipation of body-scaled action is modified in anorexia nervosa. Neuropsychologia (2010) 48(13):3961–6. 10.1016/j.neuropsychologia.2010.09.004 20833193

[13] Keizer A Smeets M Postma A van Elburg A Dijkerman HC Does the experience of ownership over a rubber hand change body size perception in anorexia nervosa patients? Neuropsychologia (2014) 62:26–37. 10.1016/j.neuropsychologia.2014.07.003 25050852

[14] Boehm I Finke B Tam FI Fittig E Scholz M Gantchev K Effects of perceptual body image distortion and early weight gain on long-term outcome of adolescent anorexia nervosa. Eur Child Adolesc Psychiatry (2016) 25(12):1319–26. 10.1007/s00787-016-0854-1 27154049

[15] Keel PK Dorer DJ Franko DL Jackson SC Herzog DB Postremission predictors of relapse in women with eating disorders. Am J Psychiatry (2005) 162(12):2263–8. 10.1176/appi.ajp.162.12.2263 16330589

[16] Roy M Meilleur D Body image distortion change during inpatient treatment of adolescent girls with restrictive anorexia nervosa. Eat Weight Disord (2010) 15(1–2):e108–15. 10.1007/BF03325289 20571314

[17] Urgesi C Fornasari L Perini L Canalaz F Cremaschi S Faleschini L Visual body perception in anorexia nervosa. Int J Eat Dis (2012) 45(4):501–11. 10.1002/eat.20982 22271579

[18] Keizer A Smeets MAM Dijkerman HC van den Hout M Klugkist I van Elburg A Tactile body image disturbance in anorexia nervosa. Psychiatry Res (2011) 190(1):115–20. 10.1016/j.psychres.2011.04.031 21621275

[19] Keizer A Smeets MAM Dijkerman HC van Elburg A Postma A Aberrant somatosensory perception in anorexia nervosa. Psychiatry Res (2012) 200(2–3):530–7. 10.1016/j.psychres.2012.05.001 22648006

[20] Guardia D Carey A Cottencin O Thomas P Luyat M Disruption of spatial task performance in anorexia nervosa. PLoS ONE (2013a) 8(1): e54928. 10.1371/journal.pone.0054928 23349990 PMC3548773

[21] Guardia D Cottencin O Thomas P Dodin V Luyat M Spatial orientation constancy is impaired in anorexia nervosa. Psychiatry Res (2012) 195(1–2):56–9. 10.1016/j.psychres.2011.08.003 21872340

[22] Badoud D Tsakiris M From the body’s viscera to the body’s image: is there a link between interoception and body image concerns? Neurosci Biobehav Rev (2017) 77:237–46. 10.1016/j.neubiorev.2017.03.017 28377099

[23] Pollatos O Kurz AL Albrecht J Schreder T Kleemann AM Schöpf V Reduced perception of bodily signals in anorexia nervosa. Eat Behav (2008) 9(4):381–8. 10.1016/j.eatbeh.2008.02.001 18928900

[24] Eshkevari E Rieger E Longo MR Haggard P Treasure J Increased plasticity of the bodily self in eating disorders. Psychol Med (2012) 42(4):819–28. 10.1017/S0033291711002091 22017964

[25] Botvinick M Cohen J Rubber hand feels touch that eyes see. Nature (1998) 391:756. 10.1038/35784 9486643

[26] Keizer A van Elburg A Helms R Dijkerman HC A virtual reality full body illusion improves body image disturbance in anorexia nervosa. Plos One (2016) 11(10):e0163921. 10.1371/journal.pone.0163921 27711234 PMC5053411

[27] Eshkevari E Rieger E Longo MR Haggard P Treasure J Persistent body image disturbance following recovery from eating disorders. Int J Eat Dis (2014) 47(4):400–9. 10.1002/eat.22219 24243423

[28] Bardone-Cone AM Harney MB Maldonado CR Lawson MA Robinson DP Smith R Defining recovery from an eating disorder: conceptualization, validation, and examination of psychosocial functioning and psychiatric comorbidity. Behav ResTher (2010) 48(3):194–202. 10.1016/j.brat.2009.11.001 PMC2829357 19945094

[29] Haggard P Sense of agency in the human brain. Nat Rev Neurosci (2017) 18(4):196–207. 10.1038/nrn.2017.14 28251993

[30] Synofzik M Vosgerau G Newen A I move, therefore I am: a new theoretical framework to investigate agency and ownership. Conscious Cogn (2008) 17(2):411–24. 10.1016/j.concog.2008.03.008 18411059

[31] Tsakiris M Schütz-Bosbach S Gallagher S On agency and body-ownership: phenomenological and neurocognitive reflections. Conscious Cogn (2007) 16(3):645–60. 10.1016/j.concog.2007.05.012 17616469

[32] Gentsch A Schutz-Bosbach S Endrass T Kathmann N Dysfunctional forward model mechanisms and aberrant sense of agency in obsessive-compulsive disorder. Biol Psychiatry (2012) 71(7):652–9. 10.1016/j.biopsych.2011.12.022 22305109

[33] Voss M Chambon V Wenke D Kühn S Haggard P In and out of control: brain mechanisms linking fluency of action selection to self-agency in patients with schizophrenia. Brain (2017) 140(8):2226–39. 10.1093/brain/awx136 28899009

[34] Guardia D Metral M Pigeyre M Bauwens I Cottencin O Luyat M Body distortions after massive weight loss: lack of updating of the body schema hypothesis. EAT WEIGHT DISORD-ST (2013b) 18(3):333–6. 10.1007/s40519-013-0032-0 23760908

[35] Keizer A Smeets MAM Dijkerman HC Uzunbajakau SA van Elburg A Postma A Too fat to fit through the door: first evidence for disturbed body-scaled action in anorexia nervosa during locomotion. PLoS ONE (2013) 8(5):e64602. 10.1371/journal.pone.0064602 23734207 PMC3667140

[36] Metral M Guardia D Bauwens I Guerraz M Lafargue G Cottencin O Painfully thin but acting inside a fatter body: emphasis of sensorimotor abnormalities in anorexia nervosa between weight loss and regain. BMC Psychiatry (2014) 7(1):1–11. 10.1186/1756-0500-7-707

[37] Kalckert A Ehrsson HH Moving a rubber hand that feels like your own: a dissociation of ownership and agency. Front Hum Neurosci (2012) 6:1–14. 10.3389/fnhum.2012.00040 22435056 PMC3303087

[38] Kalckert A Ehrsson HH The moving rubber hand illusion revisited: comparing movements and visuotactile stimulation to induce illusory ownership. Conscious Cogn (2014a) 26(1):117–32. 10.1016/j.concog.2014.02.003 24705182

[39] Exterkate CC Vriesendorp PF de Jong CAJ Body attitudes in patients with eating disorders at presentation and completion of intensive outpatient day treatment. Eat Behav (2009) 10(1):16–21. 10.1016/j.eatbeh.2008.10.002 19171312

[40] Ahern AL Bennett KM Hetherington MM Internalization of the ultra-thin ideal: positive implicit associations with underweight fashion models are associated with drive for thinness in young women. Eat Disord (2008) 16(4):294–307. 10.1080/10640260802115852 18568920

[41] Stice E Fisher M Lowe MR Are dietary restraint scales valid measures of acute dietary restriction? Unobtrusive observational data suggest not. Psychol Assess (2004) 16(1):51–9. 10.1037/1040-3590.16.1.51 15023092

[42] Vitousek KB Daly J Heiser C Reconstructing the internal world of the eating-disordered individual: overcoming denial and distortion in self-report. Int J Eat Dis (1991) 10(6):647–66. 10.1002/1098-108X(199111)10:6<647::AID-EAT2260100604>3.0.CO;2-T

[43] Vartanian LR Polivy J Herman CP Implicit cognitions and eating disorders: their application in research and treatment. Cogn Behav Pract (2004) 11(2):160–7. 10.1016/S1077-7229(04)80027-0

[44] Aspen V Darcy AM Lock J A review of attention biases in women with eating disorders. Cogn Emot (2013) 27(5):820–38. 10.1080/02699931.2012.749777 PMC3610839 23228135

[45] Robinson A Safer DL Austin JL Etkin A Does implicit emotion regulation in binge eating disorder matter? Eat Behav (2015) 18:186–91. 10.1016/j.eatbeh.2015.05.011 PMC4518854 26117164

[46] Greenwald AG McGhee DE Schwartz JL Measuring individual differences in implicit cognition: the implicit association test. J Pers Soc Psychol (1998) 74(6):1464–80. 10.1037/0022-3514.74.6.1464 9654756

[47] Richetin J Perugini M Prestwich A O’Gorman R The IAT as a predictor of food choice: the case of fruits versus snacks. Int J Psychol (2007) 42(3):166–73. 10.1080/00207590601067078

[48] Greenwald AG Farnham SD Using the implicit association test to measure. J Pers Soc Psychol (2000) 79(6):1022–38. 10.1037//0022-3514.79.6.1022 11138752

[49] Lane K. A. Banaji M. R. Nosek B. A. Greenwald A. G. Understanding and using the Implicit Association Test: IV. What we know (so far) about the method. In Wittenbrink B. Schwarz N. (Eds.), Implicit measures of attitudes. New York, NY: Guilford Press (2007) (59 –102).

[50] O’Brien KS Hunter JA Halberstadt J Anderson J Body image and explicit and implicit anti-fat attitudes: the mediating role of physical appearance comparisons. Body Image (2007) 4(3):249–56. 10.1016/j.bodyim.2007.06.001 18089271

[51] Richetin J Xaiz A Maravita A Perugini M Self-body recognition depends on implicit and explicit self-esteem. Body Image (2012) 9(2):253–60. 10.1016/j.bodyim.2011.11.002 22153802

[52] Gumble A Carels R The harmful and beneficial impacts of weight bias on well-being: the moderating influence of weight status. Body Image (2012) 9(1):101–7. 10.1016/j.bodyim.2011.07.005 21871850

[53] Preston C Ehrsson HH Implicit and explicit changes in body satisfaction evoked by body size illusions: implications for eating disorder vulnerability in women. PloS One (2018) 13(6):1–31. 10.1371/journal.pone.0199426 PMC6013093 29928005

[54] Cserjési R Vermeulen N Luminet O Marechal C Nef F Simon Y Explicit vs. implicit body image evaluation in restrictive anorexia nervosa. Psychiatry Res (2010) 175(1–2):148–53. 10.1016/j.psychres.2009.07.002 19931183

[55] Parling T Cernvall M Stewart I Barnes-Holmes D Ghaderi A Using the implicit relational assessment procedure to compare implicit pro-thin/anti-fat attitudes of patients with anorexia nervosa and non-clinical controls. Eat Disord (2012) 20(2):127–43. 10.1080/10640266.2012.654056 22364344

[56] Smith AR Joiner TE Dodd DR Examining implicit attitudes toward emaciation and thinness in anorexia nervosa. Int J Eat Dis (2014) 47(2):138–47. 10.1002/eat.22210 24488837

[57] Spring VL Bulik CM Implicit and explicit affect toward food and weight stimuli in anorexia nervosa. Eat Behav (2014) 15(1):91–4. 10.1016/j.eatbeh.2013.10.017 24411758

[58] Hagman J Gardner RM Brown DL Gralla J Fier JM Frank GKW Body size overestimation and its association with body mass index, body dissatisfaction, and drive for thinness in anorexia nervosa. Eating and Weight Disorders-Studies on Anorexia, Bulimia and Obesity (2015) 20(4):449-55. 10.1007/s40519-015-0193-0 25929983

[59] Preston C Ehrsson HH Illusory changes in body size modulate body satisfaction in a way that is related to non-clinical eating disorder psychopathology. PLoS ONE (2014) 9(1):e85773. 10.1371/journal.pone.0085773 24465698 PMC3897512

[60] Preston C Ehrsson HH Illusory obesity triggers body dissatisfaction responses in the insula and anterior cingulate cortex. Cereb Cortex (2016) 26(12):4450:60. 10.1093/cercor/bhw313 PMC5193143 27733537

[61] Groves K Kennett S Gillmeister H Evidence for ERP biomarkers of eating disorder symptoms in women. Biol Psychol (2017) 123:205–19. 10.1016/j.biopsycho.2016.12.016 28057515

[62] Mond JM Myers TC Crosby RD Hay PJ Rodgers B Morgan JF Screening for eating disorders in primary care: EDE-Q versus SCOFF. Behav ResTher (2008) 46(5):612–22. 10.1016/j.brat.2008.02.003 18359005

[63] Peirce JW PsychoPy — psychophysics software in Python. J Neurosci Methods (2007) 162(1–2):8–13. 10.1016/j.jneumeth.2006.11.017 17254636 PMC2018741

[64] Benjamini Y. Hochberg Y. Controlling the false discovery rate: a practical and powerful approach to multiple testing. J R Stat Soc (1995). Series B (Methodological), 289–300.

[65] Durkin SJ Paxton SJ Predictors of vulnerability to reduced body image satisfaction and psychological wellbeing in response to exposure to idealized female media images in adolescent girls. J Psychosom Res (2002) 53:995–1005. 10.1016/S0022-3999(02)00489-0 12445589

[66] Cahill S Mussap AJ Emotional reactions following exposure to idealized bodies predict unhealthy body change attitudes and behaviors in women and men. J Psychosom Res (2007) 62(6):631–9. 10.1016/j.jpsychores.2006.11.001 17540220

[67] Fairburn CG Beglin S Assessment of eating disorders: interview or self-report questionnaire? Int J Eat Disord (1994) 16(4):363–70. 10.1002/1098-108X(199412)16:4 7866415

[68] Luce KH Crowther JH The reliability of the eating disorder examination — self-report questionnaire version (EDE-Q). Int J Eat Disord (1999) 25: (3):349–51. 10.1002/(SICI)1098-108X(199904)25:3<349::AID-EAT15>3.3.CO;2-D 10192002

[69] Peterson CB Crosby RD Wonderlich SA Joiner T Crow SJ Mitchell JE Psychometric properties of the eating disorder examination-questionnaire: factor structure and internal consistency. Int J Eat Disord (2007) 40(2):386–9. 10.1002/eat.20373 17304585

[70] Pallant J. SPSS survival manual: A step by step guide to data analysis using SPSS for Windows. 3rd ed Berkshire: Open University Press (2007).

[71] Jenkinson PM Preston C New reflections on agency and body ownership: the moving rubber hand illusion in the mirror. Conscious Cogn (2015) 33:432–42. 10.1016/j.concog.2015.02.020 25792444

[72] Greenwald AG Nosek BA Banaji MR Understanding and using the implicit association test: I. an improved scoring algorithm. J Pers Soc Psychol (2003) 85(2):197–216. 10.1037/0022-3514.85.2.197 12916565

[73] Abdulkarim Z Ehrsson HH No causal link between changes in hand position sense and feeling of limb ownership in the rubber hand illusion. Atten Percept Psychophys (2016) 78(2):707–20. 10.3758/s13414-015-1016-0 PMC4744264 26555651

[74] Rohde M Luca M Ernst MO The rubber hand illusion: feeling of ownership and proprioceptive drift do not go hand in hand. PLoS ONE (2011) 6(6): e21659. 10.1371/journal.pone.0021659 21738756 PMC3125296

[75] Kammers MPM de Vignemont F Verhagen L Dijkerman HC The rubber hand illusion in action. Neuropsychologia (2009) 47(1):204–11. 10.1016/j.neuropsychologia.2008.07.028 18762203

[76] Pyasik M Burin D Pia L On the relation between body ownership and sense of agency: a link at the level of sensory-related signals. Acta Psychol (2018) 185:219–28. 10.1016/j.actpsy.2018.03.001 29533775

[77] Tsakiris M Haggard P Experimenting with the acting self. Cogn Neuropsychol (2005) 22(3–4):387–407. 10.1080/02643290442000158 21038257

[78] Kalckert A Ehrsson HH The spatial distance rule in the moving and classical rubber hand illusions. Conscious Cogn (2014b) 30:118–32. 10.1016/j.concog.2014.08.022 25286241

[79] Marotta A Bombieri F Zampini M Schena F Dallocchio C Fiorio M The moving rubber hand illusion reveals that explicit sense of agency for tapping movements is preserved in functional movement disorders. Front Hum Neurosci (2017) 11:291. 10.3389/fnhum.2017.00291 28634447 PMC5459911

[80] Keizer A Engel MM Bonekamp J Van Elburg A Hoop training: a pilot study assessing the effectiveness of a multisensory approach to treatment of body image disturbance in anorexia nervosa. EAT WEIGHT DISORD-ST (2018) 0(0):0. 10.1007/s40519-018-0585-z PMC6751149 30288723

[81] Carey M Kupeli N Knight RE Troop N Jenkinson PM Preston CEJ Eating Disorder Examination Questionnaire (EDE-Q): norms and psychometric properties in UK females and males. Psychol Assess (2019b) 31(7): 839–50. 10.1037/pas0000703 30802119

[82] Carey M Crucianelli L Preston C Fotopoulou A The effect of visual capture towards subjective embodiment within the full body illusion. Sci Rep (2019a) 9(1):2889. 10.1038/s41598-019-39168-4 30814561 PMC6393432

[83] Castellini G Lo Sauro C Mannucci E Ravaldi C Rotella CM Faravelli C Diagnostic crossover and outcome predictors in eating disorders according to *DSM-IV* and *DSM-V* proposed criteria: a 6-year follow-up study. Psychosom Med (2011) 73(3):270–9. 10.1097/PSY.0b013e31820a1838 21257978

[84] Crucianelli L Krahé C Jenkinson PM Fotopoulou AK Interoceptive ingredients of body ownership: affective touch and cardiac awareness in the rubber hand illusion. Cortex (2017) 104:180–92. 10.1016/j.cortex.2017.04.018 28532579

[85] Crucianelli L Metcalf NK Fotopoulou A Jenkinson PM Bodily pleasure matters: velocity of touch modulates body ownership during the rubber hand illusion. Front Psychol (2013) 4:1–7. 10.3389/fpsyg.2013.00703 24115938 PMC3792699

[86] Buhlmann U Teachman BA Kathmann N Evaluating implicit attractiveness beliefs in body dysmorphic disorder using the Go/No-Go association task. J Behav Ther Exp Psychiatry (2011) 42(2):192–7. 10.1016/j.jbtep.2010.10.003 PMC3295235 21315881

[87] Buhlmann U Teachman BA Naumann E Fehlinger T Rief W The meaning of beauty: implicit and explicit self-esteem and attractiveness beliefs in body dysmorphic disorder. J Anxiety Disord (2009) 23:694–702. 10.1016/j.janxdis.2009.02.008 19278822

[88] Boepple L Thompson JK A content analytic comparison of fitspiration and thinspiration websites. Int J Eat Dis (2016) 49:98–101. 10.1002/eat.22403 25778714

[89] Tiggemann M Miller J The internet and adolescent girls’ weight satisfaction and drive for thinness. Sex Roles (2010) 63(1–2): 79–90. 10.1007/s11199-010-9789-z

[90] Gaudio S. Dakanalis A. Fariello G. Riva G. Neuroscience, brain imaging, and body image in eating and weight disorders. In: Body image, eating, and weight. Springer, Cham (2018). p. 97-111.

[91] Gaudio S Brooks SJ Riva G Nonvisual multisensory impairment of body perception in anorexia nervosa: a systematic review of neuropsychological studies. PLoS ONE (2014) 9(10):e110087. 10.1371/journal.pone.0110087 25303480 PMC4193894

[92] Gaudio S Quattrocchi CC Neural basis of a multidimensional model of body image distortion in anorexia nervosa. Neurosci Biobehav Rev (2012) 36(8):1839–47. 10.1016/j.neubiorev.2012.05.003 22613629

