# Supplementary material for: Investigating the Components of Body Image Disturbance Within Eating Disorders
Source: Front Psychiatry. 2019 Sep 18;10:635. doi: 10.3389/fpsyt.2019.00635 (PMC6759942; doi:10.3389/fpsyt.2019.00635)
Supplement: Supplementary file 1 [file DataSheet_1.pdf]

Supplementary Material

Investigating the Components of Body Image Disturbance within Eating Disorders

Mark Carey [1*] and Catherine Preston [1]

[1] Department of Psychology, University of York, York, United Kingdom

Supplementary Material

Table S1 Additional Spearman's Rank correlations for perceptual and body satisfaction measures (N=50) (*p* values are uncorrected).

		**Ownership Score Asynch**	**Agency Score Synch**	**Agency Score Asynch**	**Prop Drift Synch**	**Prop Drift Asynch**	**Baseline Hand Misestimation**	**Post Synch Misestimation**	**Post Asynch Misestimation**	**State Body Satisfaction**	**IAT Comp**	**IAT Incomp**	**IAT D Score**
**Ownership Score Synch**	Correlation Coefficient	.258	.304*	.035	.081	.074	-.175	-.226	-.090	.003	-.287*	-.009	.324*
	p value	.071	.032	.811	.575	.607	.225	.115	.533	.984	.044	.952	.022
**Ownership Score Asynch**	Correlation Coefficient		-.009	.438**	-.020	-.159	-.403**	-.305*	-.246	.127	-.093	-.017	.107
	p value		.949	.001	.889	.269	.004	.031	.085	.378	.523	.907	.461
**Agency Score Synch**	Correlation Coefficient			.122	.020	.070	.018	.071	.070	-.038	-.127	-.091	.093
	p value			.398	.893	.628	.902	.626	.628	.791	.381	.530	.521
**Agency Score Asynch**	Correlation Coefficient				.076	-.095	-.232	.041	.118	.255	-.050	.151	.164
	p value				.602	.512	.106	.776	.412	.074	.729	.296	.255
**Prop Drift Synch**	Correlation Coefficient					.088	.250	.056	.134	.042	-.266	.072	.303*
	p value					.541	.080	.699	.353	.770	.062	.620	.032
**Prop Drift Asynch**	Correlation Coefficient						-.063	-.062	.007	-.034	-.131	-.080	-.003
	p value						.663	.669	.960	.817	.364	.580	.985
**Baseline Hand Misestimation**	Correlation Coefficient							.633**	.526**	-.195	.051	-.036	-.070
	p value							.000	.000	.175	.728	.804	.629
**Post Synch Misestimation**	Correlation Coefficient								.804**	-.088	.200	.022	-.152
	p value								.000	.543	.164	.881	.293
**Post Asynch Misestimation**	Correlation Coefficient									-.060	.155	.051	-.120
	p value									.677	.281	.723	.407
**State Body Satisfaction**	Correlation Coefficient										-.344*	.101	.455**
	p value										.014	.485	.001
**IAT Comp**	Correlation Coefficient											.542**	-.426**
	p value											.000	.002
**IAT Incomp**	Correlation Coefficient												.447**
	p value												.001

Supplementary Material

Table S2 Additional Spearman's Rank correlations for perceptual and body satisfaction measures within the ED group (N=26) (*p* values are uncorrected).

		Ownership Score Asynch	Agency Score Synch	Agency Score Asynch	Prop Drift Synch	Prop Drift Asynch	Baseline Hand Misestimation	Post Synch Misestimation	Post Asynch Misestimation	State Body Satisfaction	IAT Comp	IAT Incomp	IAT D Score
**Ownership Score Synch**	Correlation Coefficient	.139	.364	-.243	.064	.246	-.037	-.047	-.049	.254	-.323	.127	.457*
	p value	.499	.067	.233	.757	.227	.858	.819	.813	.211	.108	.538	.019
**Ownership Score Asynch**	Correlation Coefficient		-.102	.198	-.070	-.281	-.388	-.272	-.349	.159	-.103	-.063	-.009
	p value		.619	.333	.733	.164	.050	.179	.080	.437	.618	.761	.967
**Agency Score Synch**	Correlation Coefficient			.003	.184	-.021	.159	.026	.124	-.182	-.095	-.053	.213
	p value			.987	.368	.919	.438	.898	.547	.373	.646	.796	.296
**Agency Score Asynch**	Correlation Coefficient				.146	-.248	-.041	-.386	-.396*	.271	.057	.257	.123
	p value				.477	.221	.844	.051	.045	.180	.780	.206	.549
**Prop Drift Synch**	Correlation Coefficient					.160	.670**	.544**	.549**	-.058	-.474*	-.031	.443*
	p value					.435	.000	.004	.004	.778	.014	.879	.024
**Prop Drift Asynch**	Correlation Coefficient						.104	-.031	.040	.249	-.280	.007	.288
	p value						.612	.879	.848	.220	.165	.974	.154
**Baseline Hand Misestimation**	Correlation Coefficient							.728**	.763**	-.315	-.214	-.116	.144
	p value							.000	.000	.117	.294	.574	.482
**Post Synch Misestimation**	Correlation Coefficient								.913**	-.360	-.177	-.002	.187
	p value								.000	.071	.388	.991	.361
**Post Asynch Misestimation**	Correlation Coefficient									-.395*	-.157	-.062	.143
	p value									.046	.443	.764	.486
**State Body Satisfaction**	Correlation Coefficient										.132	.463*	.292
	p value										.520	.017	.148
**IAT Comp**	Correlation Coefficient											.389*	-.636**
	p value											.049	.000
**IAT Incomp**	Correlation Coefficient												.380
	p value												.056

Supplementary Material

Table S3 Additional Spearman's Rank correlations for perceptual and body satisfaction measures within the HC group (N=24) (*p* values are uncorrected).

		Ownership Score Asynch	Agency Score Synch	Agency Score Asynch	Prop Drift Synch	Prop Drift Asynch	Baseline Hand Misestimation	Post Synch Misestimation	Post Asynch Misestimation	State Body Satisfaction	IAT Comp	IAT Incomp	IAT D Score
**Ownership Score Synch**	Correlation Coefficient	.361	.256	.316	.138	-.009	-.306	-.041	-.238	-.300	-.312	-.034	.262
	p value	.083	.227	.132	.521	.966	.146	.849	.262	.154	.137	.874	.217
**Ownership Score Asynch**	Correlation Coefficient		.116	.753**	-.032	.036	-.489*	-.288	-.288	-.020	.038	.101	.179
	p value		.589	.000	.881	.868	.015	.172	.173	.927	.860	.639	.402
**Agency Score Synch**	Correlation Coefficient			.267	-.118	.204	-.135	-.181	-.271	.176	-.282	-.104	.058
	p value			.207	.583	.339	.531	.398	.201	.409	.183	.628	.788
**Agency Score Asynch**	Correlation Coefficient				-.050	.096	-.419*	-.329	-.439*	.218	-.006	.068	.141
	p value				.815	.657	.041	.117	.032	.306	.979	.751	.512
**Prop Drift Synch**	Correlation Coefficient					.089	-.281	-.317	-.250	-.212	.013	.219	.234
	p value					.680	.183	.132	.238	.321	.953	.304	.271
**Prop Drift Asynch**	Correlation Coefficient						-.228	-.197	-.081	-.057	-.184	-.211	-.250
	p value						.285	.357	.707	.793	.390	.321	.239
**Baseline Hand Misestimation**	Correlation Coefficient							.716**	.751**	-.126	.224	.013	-.261
	p value							.000	.000	.557	.293	.950	.218
**Post Synch Misestimation**	Correlation Coefficient								.829**	-.270	-.138	-.334	-.334
	p value								.000	.202	.521	.110	.111
**Post Asynch Misestimation**	Correlation Coefficient									-.410*	-.027	-.228	-.286
	p value									.047	.902	.285	.176
**State Body Satisfaction**	Correlation Coefficient										-.184	-.080	.027
	p value										.388	.709	.899
**IAT Comp**	Correlation Coefficient											.732**	.100
	p value											.000	.642
**IAT Incomp**	Correlation Coefficient												.663**
	p value												.000

Supplementary Material

Table S4 Additional Spearman's Rank correlations for perceptual measures and ED psychopathology (N=50) (*p* values are uncorrected).

		BMI	Restraint	Eating Concern	Shape Concern	Weight Concern	Global EDEQ
**Ownership Score Synchronous**	Correlation Coefficient	.044	.136	.092	-.016	-.044	.050
	p value	.765	.347	.525	.913	.764	.731
**Ownership Score Asynchronous**	Correlation Coefficient	.147	.063	-.110	-.104	-.070	-.053
	p value	.313	.666	.448	.473	.628	.716
**Agency Score Synchronous**	Correlation Coefficient	.071	.009	-.037	.122	.072	.059
	p value	.626	.951	.800	.400	.621	.685
**Agency Score Asynchronous**	Correlation Coefficient	-.018	-.042	-.054	-.066	-.071	-.060
	p value	.902	.773	.710	.648	.623	.679
**Proprioceptive Drift Synchronous**	Correlation Coefficient	.056	.169	.088	.049	.039	.081
	p value	.704	.240	.543	.735	.790	.576
**Proprioceptive Drift Asynchronous**	Correlation Coefficient	.047	.329*	.235	.120	.117	.209
	p value	.751	.020	.100	.408	.417	.146
**Baseline Hand Misestimation**	Correlation Coefficient	-.097	.145	.094	.198	.197	.184
	p value	.509	.314	.516	.169	.171	.201
**Post Synchronous Hand Misestimation**	Correlation Coefficient	-.060	.067	.067	.086	.087	.091
	p value	.684	.643	.646	.555	.549	.528
**Post Asynchronous Hand Misestimation**	Correlation Coefficient	-.132	.103	.126	.099	.094	.111
	p value	.366	.475	.383	.496	.518	.445

Supplementary Material

Table S5 Additional Spearman's Rank correlations for perceptual measures and ED psychopathology within the ED group (N=26) (*p* values are uncorrected).

		BMI	Restraint	Eating Concern	Shape Concern	Weight Concern	Global EDEQ
**Ownership Score Synchronous**	Correlation Coefficient	-.178	.135	.025	-.304	-.390*	-.098
	p value	.395	.511	.903	.132	.049	.633
**Ownership Score Asynchronous**	Correlation Coefficient	.324	.131	-.203	-.184	.004	-.040
	p value	.114	.524	.320	.369	.985	.844
**Agency Score Synchronous**	Correlation Coefficient	.265	-.150	-.136	.120	.101	-.044
	p value	.200	.466	.509	.560	.623	.830
**Agency Score Asynchronous**	Correlation Coefficient	.028	.048	.050	.039	.161	.066
	p value	.895	.814	.807	.850	.432	.750
**Proprioceptive Drift Synchronous**	Correlation Coefficient	-.001	.294	.094	.033	.141	.169
	p value	.997	.145	.646	.871	.491	.409
**Proprioceptive Drift Asynchronous**	Correlation Coefficient	.057	.236	.163	-.234	-.160	.020
	p value	.788	.246	.426	.250	.434	.923
**Baseline Hand Misestimation**	Correlation Coefficient	-.187	.271	.070	.342	.249	.299
	p value	.372	.180	.735	.087	.220	.138
**Post Synchronous Hand Misestimation**	Correlation Coefficient	-.105	.090	-.019	.195	.096	.069
	p value	.619	.662	.927	.340	.640	.738
**Post Asynchronous Hand Misestimation**	Correlation Coefficient	-.033	.044	.008	.250	.143	.101
	p value	.874	.831	.967	.219	.486	.625

Supplementary Material

Table S6 Additional Spearman's Rank correlations for perceptual measures and ED psychopathology within the HC group (N=24) (*p* values are uncorrected).

		**BMI**	**Restraint**	**Eating Concern**	**Shape Concern**	**Weight Concern**	**Global EDEQ**
**Ownership Score Synchronous**	Correlation Coefficient	.446*	.195	.176	.211	.153	.214
	p value	.029	.360	.411	.321	.475	.315
**Ownership Score Asynchronous**	Correlation Coefficient	-.172	.328	.156	.148	.028	.195
	p value	.421	.117	.467	.492	.898	.361
**Agency Score Synchronous**	Correlation Coefficient	-.148	.057	-.199	.103	.011	.051
	p value	.489	.790	.350	.633	.958	.812
**Agency Score Asynchronous**	Correlation Coefficient	-.171	.141	.168	.071	.007	.119
	p value	.424	.511	.434	.741	.976	.578
**Proprioceptive Drift Synchronous**	Correlation Coefficient	.162	.348	.431*	.341	.166	.320
	p value	.450	.095	.035	.103	.439	.128
**Proprioceptive Drift Asynchronous**	Correlation Coefficient	.165	.246	.032	.116	.115	.192
	p value	.440	.247	.881	.590	.592	.368
**Baseline Hand Misestimation**	Correlation Coefficient	.121	-.102	-.139	.008	.115	-.038
	p value	.572	.635	.517	.971	.592	.861
**Post Synchronous Hand Misestimation**	Correlation Coefficient	.262	.033	-.070	.188	.410*	.189
	p value	.217	.878	.744	.379	.046	.376
**Post Asynchronous Hand Misestimation**	Correlation Coefficient	.078	-.013	-.128	.037	.167	.034
	p value	.716	.952	.550	.863	.435	.875

Supplementary Material

Table S7 Additional Spearman's Rank correlations for body satisfaction measures and ED psychopathology (N=50) (*p* values are uncorrected).

		BMI	Restraint	Eating Concern	Shape Concern	Weight Concern	Global EDEQ
**State Body Satisfaction**	Correlation Coefficient	.019	-.620**	-.726**	-.809**	-.803**	-.794**
	p value	.895	.000	.000	.000	.000	.000
**IAT Compatible Trials**	Correlation Coefficient	-.097	.203	.223	.308*	.298*	.262
	p value	.509	.158	.119	.030	.036	.066
**IAT Incompatible Trials**	Correlation Coefficient	-.124	.077	.060	-.077	-.042	-.028
	p value	.398	.596	.677	.596	.770	.847
**IAT D Score**	Correlation Coefficient	.051	-.186	-.198	-.465**	-.409**	-.353*
	p value	.727	.196	.169	.001	.003	.012
**BMI**	Correlation Coefficient		-.229	-.131	-.028	.088	-.087
	p value		.113	.370	.851	.547	.553
**Restraint**	Correlation Coefficient			.802**	.707**	.746**	.886**
	p value			.000	.000	.000	.000
**Eating Concern**	Correlation Coefficient				.819**	.812**	.920**
	p value				.000	.000	.000
**Shape Concern**	Correlation Coefficient					.936**	.932**
	p value					.000	.000
**Weight Concern**	Correlation Coefficient						.943**
	p value						.000
**Global EDEQ**	Correlation Coefficient						
	p value						

Supplementary Material

Table S8 Additional Spearman's Rank correlations for body satisfaction measures and ED psychopathology within the ED group (N=26) (*p* values are uncorrected).

		BMI	Restraint	Eating Concern	Shape Concern	Weight Concern	Global EDEQ
**State Body Satisfaction**	Correlation Coefficient	-.161	.028	-.189	-.693**	-.538**	-.408*
	p value	.441	.893	.354	.000	.005	.038
**IAT Compatible Trials**	Correlation Coefficient	.000	-.274	-.382	-.036	-.117	-.268
	p value	1.000	.175	.054	.863	.569	.185
**IAT Incompatible Trials**	Correlation Coefficient	-.117	-.049	-.114	-.345	-.238	-.287
	p value	.578	.811	.578	.084	.243	.155
**IAT D Score**	Correlation Coefficient	.062	.183	.298	-.295	-.050	.016
	p value	.767	.372	.139	.143	.810	.937
**BMI**	Correlation Coefficient		-.252	.103	.240	.462*	.145
	p value		.224	.623	.248	.020	.489
**Restraint**	Correlation Coefficient			.494*	.027	.268	.686**
	p value			.010	.897	.185	.000
**Eating Concern**	Correlation Coefficient				.367	.542**	.807**
	p value				.065	.004	.000
**Shape Concern**	Correlation Coefficient					.806**	.644**
	p value					.000	.000
**Weight Concern**	Correlation Coefficient						.780**
	p value						.000
**Global EDEQ**	Correlation Coefficient						
	p value						

Supplementary Material

Table S9 Additional Spearman's Rank correlations for body satisfaction measures and ED psychopathology within the HC group (N=24) (*p* values are uncorrected).

		BMI	Restraint	Eating Concern	Shape Concern	Weight Concern	Global EDEQ
**State Body Satisfaction**	Correlation Coefficient	-.317	-.468*	-.263	-.402	-.353	-.398
	p value	.131	.021	.215	.051	.091	.054
**IAT Compatible Trials**	Correlation Coefficient	-.104	.183	.008	.078	.089	.075
	p value	.629	.393	.972	.716	.679	.728
**IAT Incompatible Trials**	Correlation Coefficient	-.031	.178	.192	-.044	-.023	.024
	p value	.886	.407	.369	.840	.916	.910
**IAT D Score**	Correlation Coefficient	-.010	.103	.181	-.261	-.211	-.124
	p value	.965	.634	.398	.217	.323	.563
**BMI**	Correlation Coefficient		.219	.080	.112	.316	.190
	p value		.304	.711	.601	.132	.375
**Restraint**	Correlation Coefficient			.436*	.741**	.699**	.852**
	p value			.033	.000	.000	.000
**Eating Concern**	Correlation Coefficient				.596**	.373	.631**
	p value				.002	.073	.001
**Shape Concern**	Correlation Coefficient					.841**	.949**
	p value					.000	.000
**Weight Concern**	Correlation Coefficient						.895**
	p value						.000
**Global EDEQ**	Correlation Coefficient						
	p value						
